# Teaching genetics prior to teaching evolution improves evolution understanding but not acceptance

**DOI:** 10.1371/journal.pbio.2002255

**Published:** 2017-05-23

**Authors:** Rebecca Mead, Momna Hejmadi, Laurence D. Hurst

**Affiliations:** The Milner Centre for Evolution, Department of Biology and Biochemistry, University of Bath, Bath, England; University of Maine, United States of America

## Abstract

What is the best way to teach evolution? As microevolution may be configured as a branch of genetics, it being a short conceptual leap from understanding the concepts of mutation and alleles (i.e., genetics) to allele frequency change (i.e., evolution), we hypothesised that learning genetics prior to evolution might improve student understanding of evolution. In the UK, genetics and evolution are typically taught to 14- to 16-y-old secondary school students as separate topics with few links, in no particular order and sometimes with a large time span between. Here, then, we report the results of a large trial into teaching order of evolution and genetics. We modified extant questionnaires to ascertain students’ understanding of evolution and genetics along with acceptance of evolution. Students were assessed prior to teaching, immediately post teaching and again after several months. Teachers were not instructed what to teach, just to teach in a given order. Regardless of order, teaching increased understanding and acceptance, with robust signs of longer-term retention. Importantly, teaching genetics before teaching evolution has a significant (*p* < 0.001) impact on improving evolution understanding by 7% in questionnaire scores beyond the increase seen for those taught in the inverse order. For lower ability students, an improvement in evolution understanding was seen only if genetics was taught first. Teaching genetics first additionally had positive effects on genetics understanding, by increasing knowledge. These results suggest a simple, minimally disruptive, zero-cost intervention to improve evolution understanding: teach genetics first. This same alteration does not, however, result in a significantly increased acceptance of evolution, which reflects a weak correlation between knowledge and acceptance of evolution. Qualitative focus group data highlights the role of authority figures in determination of acceptance.

## Author summary

What is the best way to teach evolution? We hypothesised that if students know the fundamental concepts of genetics, then this might help them understand evolution better. To evaluate this, we performed a large trial in which pupils in United Kingdom secondary schools were either taught genetics and then evolution or evolution and then genetics. We found that the students being taught genetics first had a 5%–10% improvement in their understanding of evolution, above that shown in the group taught evolution first. The change was seen in both higher- and foundation-ability classes. Indeed, in the foundation classes the genetics-first approach was the only approach that enabled an increase in evolution understanding. Teaching genetics first comes at no cost to genetics understanding (and may even improve it). However, the genetics-first approach was no different from the evolution-first approach in helping the acceptance of evolution. Qualitative follow-up studies indicated a major role for authority figures in determining acceptance, potentially explaining the weak correlation between understanding and acceptance. These results suggest a simple, minimally disruptive, zero-cost intervention to improve evolution understanding: teach genetics first.

## Introduction

While Dobzhansky famously wrote, “nothing in biology makes sense except in the light of evolution” [1], evolution remains one of the most misunderstood topics in biology [2–4]. Despite its importance, public understanding and acceptance of evolution is considered poor [5,6]. School-level teaching of evolution is, thus, important, at the very least because this is often potentially the first formal introduction many people have to the scientific understanding of the theory. There are, however, concerns over its unsatisfactory teaching [5,7,8]. Many studies suggest that not all teachers fully understand the theory of evolution [9–15] and that some teachers incorporate alternative nonscientific explanations within evolution lessons or even avoid the topic completely [8,10,16,17]. Students’ grasp of evolution is often poor and does not always agree with the scientific understanding [18–21]. Commensurately, numerous studies report low levels of understanding among first year undergraduate students [22–24]. These factors likely contribute to the poor public understanding of evolution reported by many researchers, including in the UK context [25,26]. This tempts the question, what are the best methods to teach evolution?

This issue here is currently much debated, particularly at the secondary school level (e.g. [27–29]). This is because the theory of evolution can be a controversial issue [8,16,30]. Strong opposition is well documented in the United States (e.g., [31–39]), but there is increasing concern about the impact that religious movements or strong cultural and social traditions may have on evolution education in other countries, including Northern Ireland [40], Poland [41], Turkey [42], and the UK [25,43]. There are also concerns that creationism has been taught in UK schools and that religious-motivated groups have attempted to influence science lessons [25,43,44]. More generally, numerous studies have focused on impediments to understanding and acceptance of evolution. While religious orientation [42,45–47], prior acceptance/rejection of the theory of evolution [46,48,49], and views of authority figures including teachers and religious leaders [16,18,48] are commonly cited reasons, reasoning skills [18,19,45,48,50,51] are also considered to be of importance. However, there has been relatively little work focused on improving teaching and understanding of evolution. Much of the current evolution education research is based on university students or teachers and may not be applicable to school students. Secondary school level biology is usually compulsory for all students up to the age of 16, whereas university level courses are likely to be taken by more scientifically and academically oriented students, therefore studies related to university students need not be very meaningful for younger learners.

Regardless of whether there is any real reason for concern, there is no research within the UK that we are aware of that investigates evolution acceptance and understanding amongst secondary school-aged students or that investigates factors that might impact understanding and acceptance. If concerns over evolution education are genuine, then research is needed to find the best way to improve the situation. If, however, there is less need for concern, UK students may provide an interesting research focus as to how best to teach evolution, which could be applicable not just within the UK but in countries where evolution acceptance is more problematic.

As microevolution may be configured as a branch of genetics, it being a short conceptual leap from understanding the concepts of mutation and alleles (i.e., genetics) to allele frequency change (i.e., evolution), we hypothesised that learning genetics prior to evolution might improve student understanding of evolution. Prior research suggests a relationship between evolution acceptance and genetics understanding, or “genetic literacy,” exists [6]. This idea of a relationship between knowledge of genetics and acceptance of evolution has not been widely studied. It seems intuitive to hypothesise that good understanding of genetics should help understanding, and possibly acceptance, of evolution. DNA is the heritable material through which variation needed for evolution occurs. Moreover, as basic genetics (as opposed to applied genetics, genetically modified [GM] crops, cloning, etc.) is a relatively neutral subject and not considered controversial in the ways that evolution might be, teaching genetics first could be a means of improving evolution education without any concerns over potentially controversial issues.

There are supporters of linking the teaching of evolution with genetics, but for the most part, this is based on opinion [28,52]. There are also some that do not view a role for genetics as helpful, noting that Darwin didn’t know about genetics [53]. However, this argument would seem counterproductive, as the study of genetics does provide further understanding of, and evidence for, the theory of evolution. Moreover, Darwin’s argument for the logic of Natural Selection in the early chapters of the *Origin of Species* specifically required a heritable component [54]. There are rare examples of studies in which trial teaching programmes include a sequential aspect [19], but overall improvements in students’ understanding of evolution and genetics may have been linked to the constructivist nature of the teaching programme, rather than the order of topics or any links made between them. Clearly, there is scope for further work related to this.

The test that we perform is based within the UK setting. Since the National Curriculum was introduced for schools in England and Wales in 1988, evolution has been a compulsory part of secondary science classes and is currently included in GCSE (General Certificate of Education) science and biology examination courses, typically taught to 14–16-year-olds [25,55]. This is when most students are first introduced to the theory of evolution during their school education. Parenthetically, this situation is changing, as evolution was introduced to the primary curriculum in 2014. This does not affect the students involved in this research study, but it will be very interesting to see if and how this earlier introduction impacts students as they progress onto secondary school and beyond.

The secondary school biology GCSE courses involved in this research project contain separate modules or topics featuring evolution and genetics. Most of these do not specifically link evolution and genetics, despite the obvious relationship between the two. According to exam board specifications and many secondary school textbooks, evolution is supported by fossil evidence, but there is rarely any mention of genetics. Students are generally taught within “higher”- or “foundation”- ability classes, and the hours of tuition and exact content studied varies between these sets. Although both genetics and evolution are taught to secondary school-aged students, the order of these topics depends on exam boards and school or teacher preference, and topics are not necessarily taught consecutively.

We hypothesise that if students learn about genetics before evolution, this simple intervention could have a positive impact on learning. Our motivation for this test is not simply one based on some limited prior data and intuition. Rather, at least within the UK context, teachers are under unprecedented pressures, be these of time, resources, or regular changes to the curriculum. For a suggested change to teaching practices to have scope to be readily adopted, we sought to find interventions that are cost free, minimally disruptive, and require no further changes to what is taught. Thus, in our trial all that was changed was the order of teaching of material; exactly what was to be taught was left to teachers’ discretion. Moreover, what they were teaching is constrained by the stipulations of the different exam boards. Our experimental design thus attempts to mimic what would happen were any new ordering to be made policy.

In brief, we asked teachers to teach in 1 of 2 orders: genetics first or evolution first. As it would be unethical to request some students not to be taught both subjects, we have no control. Our design may be considered a version of a crossover design [56]. The students were tested before being taught, immediately after and then several months after. Student response we define in 3 dimensions: understanding of evolution, understanding of genetics, and acceptance of evolution. Understanding of evolution (and genetics) refers to knowledge of a subject and practical application of this knowledge (here, evolution and genetics) and is different from acceptance, which refers to agreement with an idea or theory or the recognition that a position is valid or correct (here, the theory of evolution). The quantitative tests were modifications of accepted tests of genetics understanding, evolution understanding, and evolution acceptance. We followed up with more limited quantitative analysis from interviews with students. With a large number of classes being analysed, we presumed that our attempts to randomise which teacher teaches in which order would remove teacher, class, or cohort effects. Sample sizes for the longer-term retention are limited, and we could only assess general trends, not order effects. We noted when schools divided students between high- and foundation-ability classes and stratified with respect to this variable.

We ask first whether teaching has any demonstrable impact on understanding and whether “ability” has any effect. “Ability” is very crudely used as an estimate of intelligence, which in itself is highly debated and complex to define. Here, ability is based on whether students are set within a higher-ability class and likely to be entered for a higher-tier exam, or whether they are within a lower-ability set and entered for a foundation-tier exam. We then ask whether teaching order affects the extent of change in understanding of evolution and of genetics. Importantly, we find evidence that teaching genetics first improves the understanding of evolution, but also of genetics, more than teaching evolution first improves both understandings. Indeed, in the foundation class evolution understanding goes up only if genetics is taught first. Finally, we address the issue of whether the same intervention affects acceptance of evolution. We find no evidence that teaching order modulates increases in evolution acceptance. This possibly stems from the weak correlation between understanding of evolution and acceptance. We employed a qualitative methodology to explore possible reasons for this.

## Results

### Understanding of genetics and evolution

#### Teaching has a positive impact on knowledge of evolution and genetics

**Fig 1:**
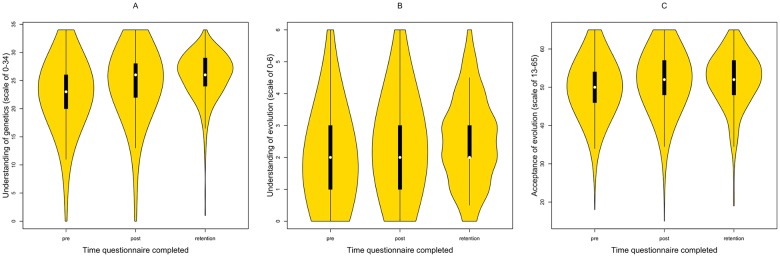
Pre-, post-, and retention test scores for A), genetics knowledge B), evolution knowledge, and C) evolution acceptance (genetics knowledge—pre: *n* = 388, post: *n* = 363, retention: *n* = 329; evolution knowledge—pre: *n* = 379, post: *n* = 346, retention: *n* = 310; evolution acceptance—pre: *n* = 388, post: *n* = 365, retention: *n* = 329). Raw data can be found in S1 and S2 Data.

If one of the purposes of education is to impart knowledge, then one would hope that teaching has a positive impact on students’ understanding of the topics being taught. We examined whether this is the case for evolution and genetics. Teaching has a positive impact on knowledge of genetics, (Fig 1A, *Z* = 177,834 *p* < .001) and on knowledge of evolution (Fig 1B, *Z* = 130,876.5, *p* < .001). Students’ knowledge of genetics increased by 2 marks (6%) on average, and by half a mark (8%) for evolution knowledge, but there was a wide range of variation. Teaching only had a positive impact on the evolution knowledge of 48% of students. 26% showed no change, and 26% showed a decrease in understanding.

#### Higher-ability students gain more knowledge of evolution

Teaching has a positive impact on evolution understanding for higher-ability students (*Z* = 95,800.5, *p* < .001) and for foundation students, (*Z* = 2,746, *p* < .001). However, higher-ability students have a greater level of evolution understanding before teaching (*W* = 223,415.5, *p* < .001) and after teaching (*W* = 169,066, *p* < .001). A linked matching pairs approach has been taken to further investigate the differences between ability groups. In this, we consider individuals of equal score in the preteaching scores, pairing those from different strata. We then consider the difference in increment in score for each pair. We then compare the values of these differential increments between the strata. By doing so, any influences that determined preteaching score are effectively eliminated as a covariate.

Both groups show a significant increase in understanding evolution (higher: *Z* = 2,888.5, *p* < .001, lower: *Z* = 2,746, *p* < .001), but there is a significant difference between the groups after teaching, with higher-ability students showing a greater understanding of evolution compared to those from foundation sets (*W* = 43761, *p* < .001), even when students start with the same understanding of evolution. ANCOVA predicting score change as a function of ability with pretest score as a covariate similarly finds an affect of ability, with higher-ability students having higher change scores (estimate 0.32, *p* = 0.002). This result might suggest that current teaching practices work best for higher-ability students.

#### Higher-ability students gain more knowledge of genetics

**Fig 2:**
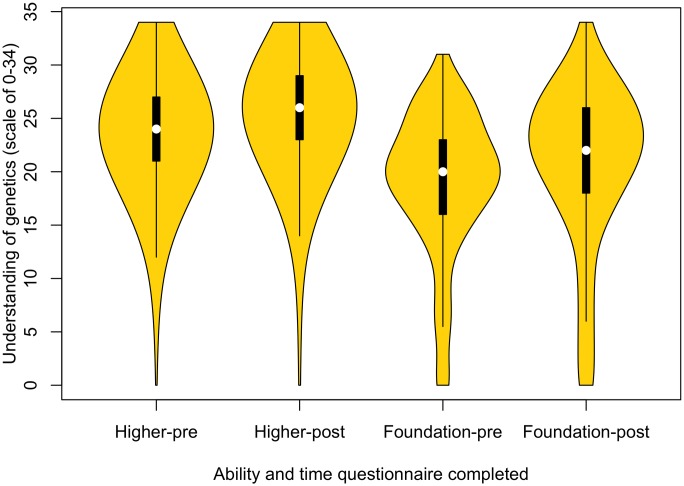
Violin plot of the understanding of genetics for higher- and foundation-ability students, pre- and post- learning about evolution and genetics (higher pre: *n* = 1,354, lower pre: *n* = 358, higher post: *n* = 1,203, lower post: *n* = 284). Raw data can be found in S1 and S2 Data.

Teaching also has a positive impact on understanding of genetics both for higher-ability students (*Z* = 114,023, *p* < .001) and for foundation students (*Z* = 6,757, *p* < .001). Again, higher-ability students have a greater level of genetics understanding before teaching, (*W* = 349,326.5, *p* < .001), and after teaching (*W* = 239,010.5, *p* < .001) (Fig 2).

A linked approach has been used again to further analyse the differences between ability groups. Both groups show a significant increase in genetics knowledge (higher: *Z* = 3,619.5, *p* < .001, lower: *Z* = 6,149.5, *p* < .001). There is a significant difference between the groups after teaching, with higher-ability students showing a greater understanding of genetics compared to those from lower-ability sets (*W* = 52,836.5, *p* < .001), even when students start with the same understanding of genetics. This is confirmed by a comparison of the change in scores for each ability group (*W* = 35,224, *p* < .001). ANCOVA predicting score change as a function of ability with pretest score as a covariate similarly finds an affect of ability (estimate 1.2, *p* <0.0001). Again, this suggests that current teaching practices work best for the higher-ability students.

#### Teaching genetics first increases evolution knowledge

**Fig 3:**
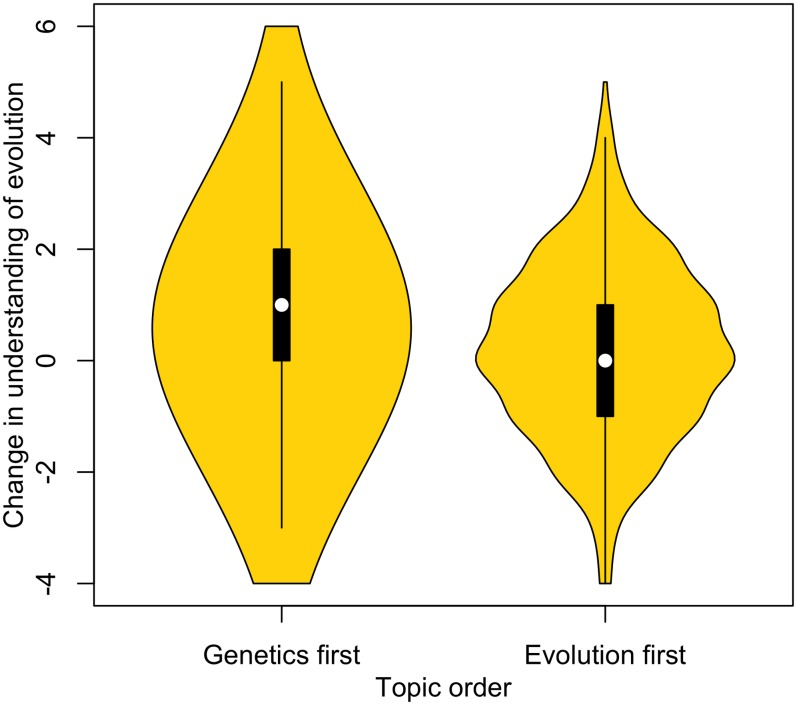
Change in understanding of evolution (i.e., after teaching − before) for different topic orders (genetics first: *n* = 779, evolution first: *n* = 454). Raw data can be found in S1 and S2 Data.

Does the teaching order—genetics or evolution first—make a difference to learning outcome? Teaching has a positive impact on evolution knowledge for those students who are taught genetics first (*Z* = 42,704, *p* < .001) and for those who are taught evolution first (*Z* = 23,566, *p* < .001). The 2 groups are not significantly different prior to learning these topics (*W* = 302,352.2, *p* = 0.5.), but those students who were taught genetics first have significantly higher post-teaching test scores than those who were taught evolution first (*W* = 267,270, *p* < .001). The change in scores was thus significantly different, with those learning genetics first showing a greater increase in evolution knowledge (*W* = 151,199.5, *p* < .001) (Fig 3). This change reflects a mean difference of 0.4 marks, representing a 7% adjustment in understanding. A linked approach was also utilised and confirmed these findings: those students who learned genetics first showed a greater increase in evolution knowledge than those who learned evolution first (*W* = 166,702.5, *p* < .001). Similarly, ANCOVA with change predicted as a function of order with pretest scores as a covariate find that genetics first improves scores most (estimate 0.35, *p* < 0.0001). We note that these *p* values are robust to multitest correction. We note too that whether or not a teacher changed their order of teaching doesn’t predict the change in evolution knowledge (*W* = 70,474, *p* = 0.25).

#### Teaching genetics first is best for improving genetics knowledge

Above, we have shown that teaching genetics first appears to lead to increased understanding of evolution. Does this come at a cost to improvement of genetics understanding? One could reasonably argue that there is no advantage to teaching genetics first if the net result is an increase in evolution knowledge but at a cost of genetics understanding. We find that, if anything, teaching genetics first increases both genetic and evolution understanding.

**Fig 4:**
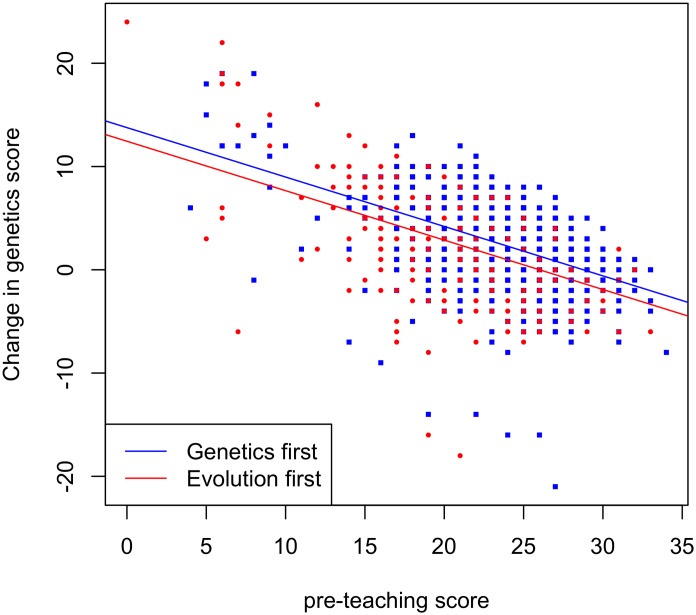
ANCOVA for change in understanding of genetics with preteaching score as a covariate for different topic orders (genetics first: *n* = 776, evolution first: *n* = 451). Here, we employ the subset of 4 pupils who completed all pre- and all postquestionanaires. Raw data can be found in S1 and S2 Data.

Those taught using either topic order show an increase in genetics knowledge (genetics first: *Z* = 63,098, *p* < .001, evolution first: *Z* = 28,941, *p* < .001). The 2 groups are, however, significantly different both before teaching (*W* = 428,985, *p* < .001) and after (*W* = 352,946.5, *p* < .001) in their genetics understanding. As they differed prior to teaching, a linked data approach was taken to compare students in the different tranches who had the same understanding prior to teaching. Again, students taught both topic orders show a significant increase in genetics understanding (genetics first: *Z* = 18,289, *p* < .001 and evolution first: *Z* = 25,277, *p* < .001), but those who learn about genetics first have significantly higher post-teaching test scores than those students who learn about evolution first (*W* = 173251, *p* < .001). The change in scores is also significantly different, with those learning genetics first showing a greater increase in genetics knowledge (*W* = 129,838, *p* < .001). The difference in change is 1.1 marks, which represents a difference of 3.5%. Allowing for differences in pretest scores via an ANCOVA (change predicted as a function of order with pretest scores as a covariate) supports the value of teaching genetics first (estimate 1.32; *p* < 0.0001; Fig 4). We note too that whether or not a teacher changed their normal order of teaching (if there was one) doesn’t predict the change in genetics knowledge (*W* = 76,988, *p* = 0.56).

### Acceptance of evolution

Having seen that teaching has a positive impact on understanding of evolution and genetics, we now consider whether teaching has an impact on acceptance of evolution. We then consider whether student ability or the order in which topics are taught have any impact on evolution acceptance.

#### Teaching of evolution increases acceptance rates

The majority of students are accepting of evolution, even before learning about the topic in secondary school. 78% of 1,712 students accept evolution before they have learned about it. Of the remaining students, 21% are undecided, and only 1% do not show acceptance towards evolution. This suggests that most students are open to learning about evolution in school (and indeed this was found within focus groups). Education has an overall positive impact on evolution acceptance. The proportion of 1,519 students who accept evolution increases to 85%, with only 14% of students undecided about evolution. 1% of students still have low acceptance. For definitions of acceptance categorisation, see Methods.

In order to better understand the changes due to teaching, acceptance scores from individual students are compared. Overall, teaching has a small but highly significant positive impact on students’ acceptance of evolution (*Z* = 175,242.5, *p* < .001) (Fig 1C). The average change in score is 2, which represents a 3% increase in acceptance. However, acceptance does not increase for all students: 2/3 demonstrate a positive change, 9% display no change, and 1/4 show a decrease in acceptance score. Not all of these changes involve marked differences, but it might be noteworthy that such a large proportion of students show some decrease in acceptance. Overall, we see that students that show larger increases in understanding of both genetics and evolution show larger increases in evolution acceptance (Spearman correlations: change in acceptance versus changes in genetics understanding: *R*_*s*_ = 0.1, *p* < 0.0001; change in acceptance versus changes in evolution understanding: *R*_*s*_ = 0.07, *p* = 0.013). There is no significant difference in the strength of these correlations (Monte Carlo simulation, *p* = 0.39). This supports the view that acceptance follows from increased understanding.

#### Higher-ability students have greater acceptance of evolution

**Fig 5:**
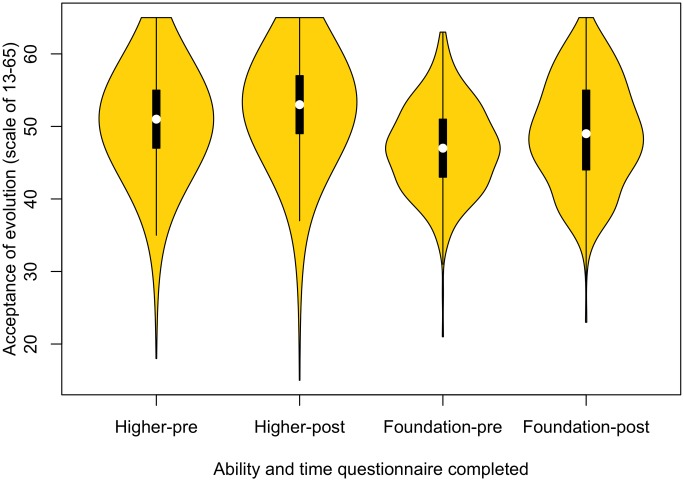
Acceptance of evolution for higher- and foundation-ability students, before and after learning about evolution and genetics (higher pre: *n* = 1,354, foundation pre: *n* = 358, higher post: *n* = 1,203, foundation post: *n* = 284). Raw data can be found in S1 and S2 Data.

Teaching has a positive impact on acceptance of evolution for both higher-ability students (*Z* = 114,312, *p* < .001) and foundation-ability groups of students (*Z* = 6,374.5, *p* < .001). However, higher-ability students had a greater level of evolution acceptance before teaching than foundation-ability students (*W* = 325,619.5, *p* < .001), as well as after teaching (*W* = 240,955.5, *p* < .001) (Fig 5).

Whether teaching makes a difference to acceptance that is different between the high- and foundation-ability students isn’t so clear. As these 2 groups were significantly different before teaching, a linked data approach was taken to further analyse a subsample of these data. Both groups show a significant increase in acceptance after teaching (higher: *Z* = 4,765, *p* < .001, lower: *Z* = 6,337.5, *p* < .001). While there is a significant difference between the groups after teaching with higher-ability students showing a greater acceptance of evolution, compared to those from lower-ability sets (*W* = 58,036, *p* = .03), this result is marginal and is sensitive to Bonferonni correction. Similarly, an effect of ability in score change is not found within an ANCOVA, in which change in score is predicted by ability controlling for pretest score (*p* for effect of ability, = 0.54).

#### Topic order has no impact on evolution acceptance

Having shown that teaching order affects evolution understanding and that teaching increases evolution acceptance, we now ask whether teaching order affects evolution acceptance as would seem a logical corollary of these 2 prior results. Students taught evolution first and students taught genetics first both showed significant increase in evolution acceptance after teaching (genetics first: *Z* = 67,718, *p* < .001, evolution first: *Z* = 25,183.5, *p* < .001). However, these initial comparisons also revealed that the 2 groups were significantly different before they learned about evolution and genetics (*W* = 377,746.5, *p* = .005), but not significantly different after learning about these topics (*W* = 289,152.5, *p* = .07). The reasons for this are unknown. A linked data approach was taken to compare students who had the same acceptance prior to teaching. There was no significant difference found between the different topics after teaching (*W* = 168,338.5 *p* = .667), and the change in acceptance score is not predicted by teaching order in an ANCOVA (*p* = 0.85).

#### Acceptance of evolution is more strongly correlated with genetics understanding than evolution understanding

Given that teaching improves evolution acceptance and understanding, and teaching order makes a difference to evolution understanding, it is curious that we detect no order effect on evolution acceptance. One possible reason for this is that the correlation between evolution understanding and acceptance is weak, and hence a small difference in understanding (owing to topic order) translates into such a small incremental difference in acceptance as to be beyond our resolution, even with comparatively large sample sizes. We thus ask about the correlation between acceptance and understanding.

Even before teaching, there is a moderate, positive relationship between evolution acceptance and genetics understanding (*R*_*s*_ = 0.42 *p* < .001). Importantly, there is a strikingly weaker positive relationship observed between evolution acceptance and evolution understanding (*R*_*s*_ = 0.24 *p* < .001) and between knowledge of genetics and knowledge of evolution (*R*_*s*_ = 0.19, *p* < .001). The correlation between genetics understanding and evolution acceptance is significantly stronger than that between evolution understanding and evolution acceptance (Monte Carlo simulation, *p* < 0.0001). We conclude that, perhaps surprisingly, genetics understanding correlates better with evolution acceptance than evolution understanding does.

In order to better understand the part knowledge plays in acceptance, partial correlations were calculated for the 2 principal variables. The correlation between evolution acceptance and genetics knowledge, given understanding of evolution, is rho = 0.39. The correlation between evolution acceptance and evolution knowledge, controlling for genetics knowledge, is only 0.18. Both results are highly significant (*p* < .001). Again, the correlation between genetics knowledge and evolution acceptance appears to be stronger than that between evolution knowledge and evolution acceptance.

**Fig 6:**
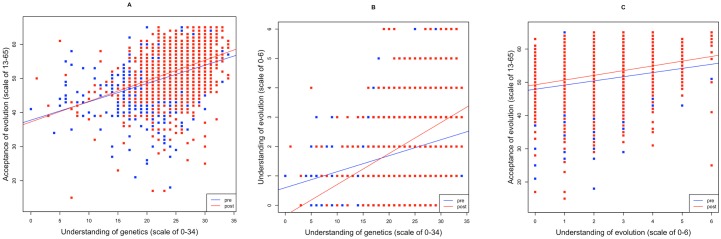
**A**. Relationship between acceptance of evolution and understanding of genetics, before and after learning about evolution and genetics. **B**. Relationship between understanding of evolution and understanding of genetics, before and after learning about evolution and genetics. **C**. Relationship between acceptance of evolution and understanding of evolution, before and after learning about evolution and genetics. Here, we employ the subset of data for pupils who completed all pre- and all postquestionanaires. Regression lines are for indicative purposes alone. Raw data can be found in S1 and S2 Data.

Correlations after teaching between evolution acceptance, genetics understanding, and evolution understanding all appear similar to those seen prior to teaching. There is significant, robust positive correlation between acceptance of evolution and knowledge of genetics (*R*_*s*_ = 0.41 *p* < .001), between acceptance of evolution and knowledge of evolution (*R*_*s*_ = 0.27 *p* < .001), and between understanding of evolution and knowledge of genetics (*R*_*s*_ = 0.39 *p* < .001) after teaching (Fig 6) (Correlations of all pre- and postrelationships can be found in Table 1). Partial correlations, again, show very similar correlations as seen previously: the correlation between evolution acceptance and genetics knowledge, controlling for evolution knowledge, is stronger than that between evolution acceptance and evolution knowledge (Table 2). The correlation between genetics understanding and evolution acceptance is again significantly stronger than that between evolution understanding and evolution acceptance (Monte Carlo simulation, *p* < 0.001).

**Table 1:** Spearman correlations between evolution acceptance, genetics knowledge, and evolution knowledge. All correlations are highly significant (*p* < .001). These pretests were done with data where students had answered all 3 preteaching questionnaires (*n* = 1,610). For the post-test scores, we again required all 3 assessments to be completed (*n* = 1,397). Raw data can be found in S1 and S2 Data.

*R*_*s*_	Pre	Post
**Evolution acceptance and genetics knowledge**	0.42	0.41
**Evolution acceptance and evolution knowledge**	0.24	0.27
**Evolution knowledge and genetics knowledge**	0.19	0.39

**Table 2:** Partial Spearman correlations between evolution acceptance and genetics knowledge, controlling for evolution knowledge and evolution acceptance and evolution knowledge, controlling for genetics knowledge. All correlations are highly significant (*p* < .001). Raw data can be found in S1 and S2 Data.

*R*_*s*_	Pre	Post
Evolution acceptance and genetics knowledge, given evolution knowledge	0.39	0.35
Evolution acceptance and evolution knowledge, given genetics knowledge	0.18	0.14

These results suggest that a unit increase in evolution understanding is likely to have only a very small incremental effect on evolution acceptance as the correlation between the 2 is weak both before and after teaching. Thus, it is logically possible that a small increment to evolution understanding (owing to order effects) translates as only a very small increase in the weakly-correlated evolution acceptance, too weak to be detectable by us. It is notable that the strongest relationship is seen between evolution acceptance and genetics knowledge. While causality here is unclear and may reflect little more than underlying ability, this we highlight as a most enigmatic result. It also lays the basis for the hypothesis that better understanding of genetics may be an optimal strategy, if the desired end is increase in both evolution understanding and evolution acceptance. This, we suggest, is worthy of further scrutiny.

#### Qualitative analysis suggests authority conditions acceptance

Our results show the positive impact on evolution understanding that teaching has and factors that impact this—most importantly, topic order. Teaching also has a positive impact on acceptance. Why, then, might acceptance and understanding be so poorly correlated? Qualitative data collected from focus groups suggest that not what is taught, but who evolution is taught by, is more important for acceptance. Many students were happy to admit that they accept evolution because it’s what they had been told by their parents or taught by their teachers, although a few students acknowledged that this is something most people tend to do when they are young:

“Because that’s what we’ve been told […] that’s what I believe.”

—(Year 10 student)

“I was told by my parents when I was younger because you believe what your parents say when you’re young.”

—(Year 10 student)

“I don’t think that we’re being lied to at all […] I just don’t think it’s like a conspiracy theory.”

—(Year 9 student)

“I don’t know if there is such evidence [for evolution], we’ve just been told that this is right.”

—(Year 9 student)

Important figures were not just people that students knew. Television documentaries were commonly given as a source of information about evolution, and some students felt these, and their presenters, were important in helping them accept evolution:

“Even in like TV programmes they make it really convincing as well.”

—(Year 11 student)

“Well, if David Attenborough believes in it then why shouldn’t everyone else?”

—(Year 10 student)

The use of “belief” as opposed to “accept” is common among students, perhaps indicating lack of understanding of differences between science and religion. The importance of religious beliefs was clearly important for some students, with some envisaging that they should pick “either one or the other.” But here, knowledge of authority figures within their religion was also important:

“I’m religious so that’s why I kind of find it difficult […] the first lesson I kind of—I kind of wanted to argue that actually God put everyone there but then I kind of also believe in science a lot so I was really confused the first lesson but—but then Miss was like ‘Oh yeah, the Pope actually agrees with some of the theories behind it’ so I kind of accepted it but don’t strongly believe in it […] I kind of just accepted because the Pope kind of believed in some of the theories that I could as well.”

—(Year 10 student)

This student had initially felt uncomfortable about learning evolution due to their religious beliefs, but having the knowledge that their religion accepted evolution was of importance. It ensured that they were able to learn about evolution and not cause any disruption to the class. It could be argued that there are underlying issues here and that, ideally, students should have a better understanding of the nature of science and its differences from religion. However, if this simple teacher intervention can ensure religious students are more comfortable and open to learning about evolution, this may appear a defensible approach to take.

### Teaching genetics first improves understanding for students of all abilities

Given the key findings regarding topic order, combined with the strong evidence found that ability has a big impact on evolution acceptance and evolution and genetics understanding, it is important to ask whether the topic order effect observed in the previous section is independent of ability. This is for 2 reasons. First, there is the purely statistical concern that if high-ability students show more improvement and more of such students learned genetics first, the trends seen need not be explained in terms of order effects. Second, teaching high-ability students is often not so much of a challenge, while finding effective mechanisms to teach the lower-ability classes can be challenging. To address both these issues, we analyse the order effects stratified by ability.

A comparison of the proportion of higher- and foundation-ability students within the 2 topic orders can be found in Table 3. From this, it is clear that, for reasons unknown, foundation-ability students tended to be taught evolution first. To address the statistical concern as well as the pedagogical concern, analysis is therefore needed to distinguish the impact of topic order from that of ability. The different topic orders for both higher- and foundation-ability groups have therefore been compared.

**Table 3:** Proportions of higher- (*n* = 1,456) and foundation- (*n* = 430) ability students taught genetics first and evolution first. Raw data can be found in S1 and S2 Data.

	Higher Ability		Foundation Ability	
	*n*	%	*n*	%
**Genetics first**	933	64	212	49
**Evolution first**	523	36	218	51

#### Teaching genetics first is best for increasing evolution knowledge for both higher- and foundation-ability students

**Fig 7:**
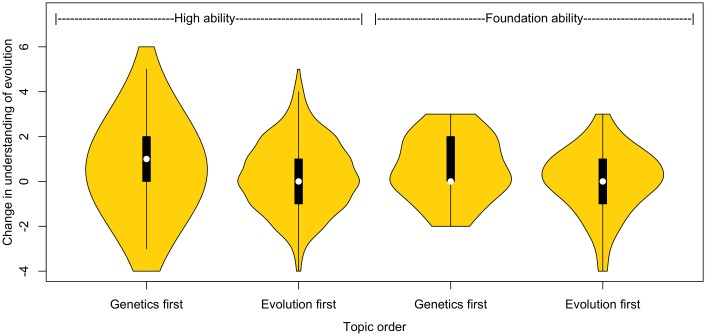
Change in understanding of evolution due to teaching for higher-ability students taught genetics first (*n* = 683) and evolution first (*n* = 372) and for foundation-ability students taught genetics first (*n* = 93) and evolution first (*n* = 79). Data plotted here is for the subset of the data in which all students answered all pre- and postquestionnaires. Raw data can be found in S1 and S2 Data.

Higher-ability students show significant increase in evolution understanding regardless of which topic order they are taught first (genetics first: *Z* = 33,239.5, *p* < .001, evolution first: *Z* = 15,882.5, *p* = .004). The 2 order groups were not significantly different before teaching (*W* = 195,495.5, *p* = .7), but higher-ability students who learned about genetics first demonstrate greater evolution knowledge after teaching than those who were taught evolution first (*W* = 1,777,056.5, *p* = .005). Similarly, there is a significant difference between the change in scores, with those taught genetics first showing the greater increase in knowledge of evolution (Fig 7.; *W* = 178,140, *p* = 1.4 x 10^−5^). The difference in change was, on average, a 6.4% increase in knowledge of evolution.

Perhaps most striking, we find that within the foundation-ability students, only those that were taught genetics first saw a significant increase in evolution understanding (genetics first: *Z* = 599, *p* < .001, evolution first: *Z* = 758, *p* = 0.9). Those who learned about genetics first showed a greater increase in understanding compared to those who were taught evolution first (Fig 7; *W* = 24,172, *p* = 0.025, sensitive to Bonferonni correction). This represents a difference of about 9% in change in scores.

In addition, we performed a multivariate regression to predict change in evolution, understanding scores with ability and topic order as factors, and pretest score as a covariate. This reveals that both topic order (estimate 0.34, *p* < 0.001) and ability (estimate 0.28, *p* = 0.0065) are predictors, as discussed above, with genetics first being beneficial.

#### Teaching genetics first increases genetics knowledge for higher- and foundation-ability students

As mentioned before, it is important to ascertain whether the teaching of genetics first comes at a cost to poorer increased understanding of genetics. We find that teaching genetics first appears to improve genetics understanding for both ability groups. Higher-ability students show a significant increase in genetics understanding regardless of which topic order they are taught first (genetics first: *Z* = 45,985, *p* < .001, evolution first: *Z* = 15,202.5, *p* < .001). However, the 2 groups are significantly different before (*W* = 263,571.5, *p* < .001) and after (*W* = 222,113.5, *p* < .001) teaching. Therefore, a linked data approach has been utilised to further investigate these changes. Again, both linked groups show a significant increase in knowledge (genetics first: *Z* = 10,050, *p* < .001, evolution first: *Z* = 13,372, *p* < .001). There is a significant difference between the 2 groups after teaching (*W* = 91,467.5, *p* < 0.001) with those who learned about genetics first having higher post-teaching scores than those who were taught evolution first. Those who learned genetics first also show a greater increase in knowledge than those taught evolution first (*W* = 70,724.5, *p* = .002), supported also by ANCOVA (estimate 1.2, *p* = 1.4 x 10^−7^).

Foundation-ability students also show a significant increase in genetics understanding regardless of which topic order they are taught first (genetics first: *Z* = 1,342, *p* < .001, evolution first: *Z* = 2,099.5, *p* < .001). The 2 groups were not significantly different before teaching (*W* = 16,048.5, *p* = .97), but foundation-ability students who learned about genetics first demonstrated greater genetics knowledge than those who were taught evolution first (*W* = 12,162, *p* = .002). There was a significant difference between the change in scores with those taught genetics first showing the greater change in knowledge of genetics (*W* = 7,933.5, *p* = .03; sensitive to multitest correction) and is supported by ANCOVA (estimate 2.0, *p* = 0.006). The average increase represents a 5% increase in knowledge of genetics.

In addition, we performed a multivariate regression to predict change in genetics understanding scores with ability and topic order as factors and pretest score as a covariate. This reveals that both topic order (estimate 1.3, *p* < 0.0001) and ability (estimate = 1.2, *p* = 0.0001) are predictors as discussed above, with genetics first being beneficial.

#### Topic order has no impact on evolution acceptance in both ability groups

Above, we found no significant effect of topic order on evolution acceptance. Does this apply to both ability groups? Higher-ability students taught either topic order show a significant increase in evolution acceptance (genetics first: *Z* = 47,143.5, *p* < .001, evolution first: *Z* = 14,699, *p* < .001). The 2 groups are significantly different before learning about evolution and genetics (*W* = 228,160, *p* = .01) but are similar after teaching (*W* = 179,555, *p* = .05). A linked approach is again used and again finds that both groups show a significant increase in knowledge after teaching (genetics first: *Z* = 12,390.5, *p* < .001, evolution first: *Z* = 14,552, *p* < .001). There is no significant difference between the post-teaching scores of the 2 groups (*W* = 91,474, *p* = .06), nor is there any difference in the amount of change between the 2 topic orders (*W* = 78,153, *p* = .06).

Similarly, foundation-ability students show a significant increase in acceptance of evolution, regardless of topic order (genetics first: *Z* = 1,822, *p* < .001, evolution first: *Z* = 1,370, *p* < .001). The 2 groups are not significantly different before (*W* = 14,718.5, *p* = .1) or after (*W* = 10,405.5, *p* = .05) teaching. There was also no significant difference in the amount of change between the 2 order groups (*W* = 6,856.5, *p* = .1). In addition, we performed a multivariate regression to predict change in evolution acceptance scores with ability and topic order as factors and pretest score as a covariate. This reveals that neither topic order (*p* < 0.88) nor ability (*p* = 0.55) are predictors.

In summary, findings related to topic order are not due to covariance with ability of students and, crucially, teaching genetics first helps both ability groups with no detriment to genetics understanding. That is to say, teaching genetics first increases understanding of evolution and genetics for both higher- and foundation-ability students. Topic order appears to have no impact on evolution acceptance, regardless of academic ability.

### Teaching has long lasting effects on acceptance and understanding

The previous results have all compared understanding and acceptance immediately before and after students have learned about evolution and genetics. In order to better understand what happens after time has lapsed since teaching, we ask whether students retain this knowledge and level of acceptance. This is an important consideration: if students show an increase in knowledge immediately after teaching, but then return to their preteaching level a few months later, it could be argued that education is not successful. Unfortunately, our sample size is too low to say anything meaningful about whether topic order has any effects on longer-term retention or to stratify this by ability.

These data are purely a subsection of all data: pre- and postdata are only included for classes that completed a retention test (therefore, numbers and statistical tests may vary slightly from results reported previously). The timing of the retention test varied between classes but was generally 3 to 6 months after these topics were taught, and did not correspond to any revision or examination of evolution or genetics. For many students, secondary school may be the last (if not only) time they learn about evolution. The responses found within these retention tests may therefore be representative of students’ lasting thoughts and understandings of these topics.

Although a smaller cohort, the subsection of students who completed the questionnaire retention test are not significantly different from the larger student sample whose questionnaire results are reported previously. They also show the same trend of significant increases in acceptance and understanding immediately after teaching, as were seen in students sampled previously, and are thus thought to be representative of the larger sample.

Teaching has a positive and long-term impact on evolution acceptance, genetics understanding, and evolution understanding. As previously observed, students show significant increase in acceptance and understanding immediately after learning about evolution and genetics (evolution acceptance: *Z* = 8,749.5, *p* < .001; genetics understanding: *Z* = 8,574.5, *p* < .001; evolution understanding: *Z* = 8,269, *p* < .001). Perhaps surprisingly, evolution acceptance and evolution understanding have not changed significantly by the time of the retention test (evolution acceptance: *Z* = 16,879, *p* = .63; evolution understanding: *Z* = 7,479; *p* = .054). Genetics understanding decreased significantly between the post-teaching and retention tests (*Z* = 12,560.5, *p* < .048). However, understanding at the point of the retention test is still significantly higher than that prior to teaching (*Z* = 5,196.5, *p* < .001). These results are all shown in Fig 1.

### No evidence for intraclass underdispersion (teacher effects)

Above, we have performed all analyses at the level of the student, ignoring possible nonindependence between data owing to the fact that the class has the same teacher (although this wasn’t always true). If any such effect is confounding our analysis, we should see lower intraclass variation in changes in scores than expected. In the most extreme circumstance, we expect all students under an “excellent” teacher to show equally large changes in scores, while those under a less competent teacher would show equally little changes in scores. Thus, in both incidences, at the limit, the intraclass variance in the change in scores would be zero.

To investigate this possibility, we calculate for each class (with more than 2 students reporting before and after data) the normalised intraclass variance in change. As we are concerned with the magnitude of the variation independent of the between-class variation in mean change, we normalise by the intraclass mean to calculate the dispersion index, which equals variance within class in change divided by mean class change. The overall reporting statistic is the mean of all the per-class dispersion index values.

To determine whether the observed mean dispersion is lower than expected, we perform a nonparametric Monte Carlo simulation. We then take each student again and randomly allocate a change score, the values being taken from those used to calculate the real mean dispersion. The random allocation is done without replacement, so each change score is used once in the real data and once in each randomization. For each Monte Carlo simulation, we then repeat the above analysis to generate a mean dispersion for all pseudoclasses (note that class structure is maintained intact, thus also controlling for class size effects).

If there were a teacher-effect causing nonindependence between data (i.e., under dispersion), we expect to see a lower mean dispersion in the real data than in randomized data (at the limit, all students within any given class go up or down to the same degree, dispersion = 0 as variance = 0). The significance of any reduction will be given by *p* = (*n*+1)/(*m*+1), where *n* is the number of randomizations in which the randomized dispersion is lower than or as low as that observed in the real data (i.e., a 1-tailed expectation). We ran each simulation 10,000 times for each of the 3 scores measures. In no case did we observe anything even approaching a significantly lower mean dispersion than expected by chance (evolution acceptance, *p* = 0.77; evolution knowledge, *p* = 0.56; genetics knowledge, *p* = 0.22). To check that this wasn’t an artefact of high variance when there are few recorded samples, we reanalysed requiring a minimum of 6 change calculations per class. We again find no evidence for underdispersion (evolution acceptance, *p* = 0.78; evolution knowledge, *p* = 0.58; genetics knowledge, *p* = 0.17). Analysing each of the 3 questions stratified into high and low groups similarly finds no evidence for underdispersion (even though the test is 1-tailed and not multitest corrected, Pmin = 0.12, Pmax = 0.82). We conclude that we see no evidence that dispersion within classes is lower than expected under a null random model and thus consider both that we see no teacher-effects and that it is appropriate to analyse at the level of the student.

### Class-level analysis supports the utility of teaching genetics first

While the above result indicates that analysis at the student level is appropriate, it is also helpful to ask whether a much more conservative class-level analysis provides any support for the hypothesis that teaching genetics first improves evolution understanding. To address this, we calculated for each class a mean change (post score − prescore) for each of the 3 parameters (acceptance and understanding x 2). We then performed Mann Whitney *U* tests for each of the 3 parameters, comparing those classes taught evolution first with those taught genetics first.

Even though the sample sizes are much more restricted than in the student-focused analysis (just 70 classes in which we have at least 1 student doing all the pre tests and all the post tests), nonetheless we see that teaching genetics first significantly improves evolution understanding (*p* = 0.004), with the mean class increase in score being approximately double that of the evolution-first treatment (Table 4). Stratifying this by ability leads to an even more conservative test, as we have only 18 qualifying foundation-ability classes. Even in this stratification, however, we still see that teaching genetics first improves evolution understanding compared to the opposite order, although the effect is significant only in the larger sample size high-ability class (*p* = 0.006), where “genetics-first” changes in scores are over 2.5 times greater than with evolution-first (Table 4). In the foundation-ability class, evolution understanding change scores are improved in the genetics-first order, but not significantly so. This may be a sample size issue, as with only 9 classes in each partition we couldn’t likely detect a 5%–10% effect even if there were one. We see no evidence that genetics understanding is adversely affected by being taught first (Table 4) and, again, no evidence that evolution acceptance is affected by ordering (Table 4).

**Table 4:** Class-level analysis of change in 3 scores as a function of teaching order. *p*-Value is from a Mann Whitney *U* test. Ea = Evolution acceptance, gk = genetics knowledge, ek = evolution knowledge. EF = evolution first, GF = Genetics first. *p*-Values in bold are significant after Bonferonni correction. Raw data can be found in S1 and S2 Data.

Variable	Ability	*p*-value	Mean change: GF	sem	*N*	Mean change: EF	sem	*N*
**ea**	all	0.293	2.094	0.304	44	2.741	0.483	26
**gk**	all	0.971	2.076	0.283	44	1.882	0.344	26
**ek**	all	**0.004**	0.614	0.081	44	0.309	0.133	26
**ea**	F	0.387	1.959	1.092	9	3.485	1.078	9
**ea**	H	0.815	2.128	0.274	35	2.347	0.47	17
**gk**	F	0.2	3.071	0.857	9	1.345	0.668	9
**gk**	H	0.447	1.82	0.271	35	2.167	0.388	17
**ek**	F	0.329	0.521	0.217	9	0.443	0.347	9
**ek**	H	**0.006**	0.638	0.087	35	0.238	0.096	17

## Discussion

Our results suggest that, at least in the UK setting, teaching of evolution and genetics is in a constructive position. Teaching improves understanding and acceptance, these increases showing strong signs of longer-term retention. Overall, evolution acceptance is high among the diverse secondary school students that we sampled, increasing to 85% after teaching. This being said, some students spoke in the focus groups of expecting there to be more evidence for evolution than was presented in their lessons, this suggesting possible improvements.

Most importantly, topic order is implicated in increasing understanding of these topics: students who are taught genetics before evolution have significantly greater knowledge of evolution and genetics, compared to students who are taught evolution first. Importantly, this same strategy appears not to be to the detriment of genetics understanding, which, if anything, is also improved by teaching genetics first. This suggests a simple, cost-free, minimally disruptive process to improve evolution understanding: namely, teaching genetics first. Importantly, too, for the foundation-ability students, only if genetics was taught first do we see an improvement in evolution knowledge. Given that there is so little cost in such a change, even if the results of our analysis were to prove not to be replicable, we can see little-to-no downside of this small switch in teaching practice. Given that teaching genetics first appears to be the only strategy that improves evolution understanding for those of foundation ability, we suggest that it might be negligent not to adopt this practice. Given, too, the strong correlation between genetics understanding and evolution acceptance, an optimal method to improve understanding of genetics and evolution, as well as to improve evolution acceptance, may be to concentrate more effort into the teaching of the genetic concepts that underpin the mechanisms of evolutionary change.

### Why might order matter?

The above results fit within the broader context of notions that order is important for learning, be that student learning or artificial intelligence learning [57], and that the importance of such effects is underappreciated [57], not least because of the dearth of empirical evidence [57]. Our study design was not focused on understanding why the order might help so much as whether the order helps. Nonetheless, we can speculate. The ordering effects could be owing to conceptual priming [58], the notion that exposure to one concept makes one more or less receptive to a subsequent stimulus, although there may well be more than 1 underlying mechanism [59]. This then would be different from the rationale as to why the ordering of the use of analogy works, as this appears rather to be owing to analogical mapping [60], not conceptual priming. While our motivation for this project was largely inspired by the possibility of conceptual priming, a possibly synergistic explanation might be that, as nearly all of the material needed to understand allele frequency change is front loaded under the banner of genetics and not under the banner of evolution, the core foundational concepts were absorbed in a context that mitigates belief-associated cognitive biases (e.g., confirmation bias [61]). That is to say, the foundational concepts (DNA, mutation, inheritance, alleles) are uncontroversial and should be easily absorbed when presented under the umbrella of genetics. Once accepted, the cognititive dissonance [62] associated with processing information under the banner “evolution” might be considerably lessened, as the student has only one bit of information to process—alleles can change frequency—which, additionally, follows as a logical consequence (conceptual priming) of the material absorbed without confirmation bias. Consistent with this, we found in qualitative data numerous examples of students anxious about learning evolution because of their religious beliefs, indicative of possible cognitive dissonance.

If the second explanation is correct, it might be best to teach material that is evolution under the banner “genetics” or “population genetics.” This “evolution by stealth” strategy could be tested by presenting the same “genetics first, evolution second” material under different banners. In the control, the students would be told explicitly that they are now switching to studying “evolution” and in the second, they would be told explicitly that they are now switching to studying “population genetics.” If the result is owing to any of the above cognitive biases (rather than something peculiar about the classroom situation), then the same “genetics first” intervention may well be profitable if employed at different educational levels (e.g., first year university level) and also in public engagement.

### Why is evolution understanding and acceptance weakly coupled?

Teaching evolution and genetics results in a significant increase in evolution acceptance, regardless of teaching order. While the “genetics first” option had demonstrable effects on evolution understanding, the same was not true for evolution acceptance. This could be explained (in statistical terms) as a consequence of a decoupling of evolutionary knowledge from acceptance of evolution. After partial correlation control, we report a correlation between evolution understanding and evolution acceptance of R = 0.18, making R^2^ just ~3% (Table 2). That is to say, variation in one parameter explains only 3% of the variation in the other. As a consequence, assuming a causal arrow between understanding and acceptance, a small but detectable increase in evolution understanding owing to teaching order is expected to have, at most, a miniscule effect on increased evolution acceptance, so miniscule as to be beyond the resolution of this study, despite its relatively large sample sizes.

Our qualitative analysis suggests coherent reasons for the decoupling of evolution understanding, not least of which is what might be considered “authority.” For students of a religious background, this appeared to be of some importance, as might be expected given the prior literature on religious-based impediments to acceptance of evolution in numerous contexts. Anecdotally, it appears that simple statements concerning the acceptance of evolution by key religious authority figures, prior to teaching, may enable some students to approach evolution as they would any other science. However, the data here are anecdotal.

The dislocation between acceptance and understanding also underscores the need to consider acceptance and understanding/knowledge as 2 distinct parameters. In addition, in this context we note that although the terms knowledge and understanding have been used synonymously, there is a difference. While students might have knowledge of a topic, they may not really understand it. Perhaps a better distinction between these terms would provide more insight into relationships with acceptance. Discussions with students would suggest that they know evidence for evolution exists, yet few are able to describe any of this evidence in any detail or even give examples. They appear to have limited understanding of evidence. However, these students are still accepting of evolution. This also leads to the question, what is known? Are there particular areas of genetics or evolution knowledge that provoke evolution acceptance? Or, indeed, are there specific aspects of genetics knowledge that enrich evolution knowledge?

### Are we teaching foundation classes appropriately?

While teaching genetics first was the only order in which the foundation students improved understanding of evolution, one possible source of concern in our results is the consistency with which teaching improves scores for higher-ability students more than it does for foundation-ability students. In some cases, we see that teaching possibly has a negative impact on foundation-ability students. Some of the latter may be owing to stochastic fluctuation and is, thus, not of concern. We also cannot fully eliminate the possibility of the bored student. However, the greater response of the high-ability students cannot be easily dismissed as, if anything, we expect there to be a “ceiling effect” of the questionnaire, in which those that demonstrate high acceptance or knowledge prior to teaching cannot increase all that much (put differently, you can’t score more than 100%).

One hypothesis to address these observations is that, despite the fact that “genetics first” was the only strategy that led to improvements of evolution understanding for the foundation-ability group, what works for the teaching of high-ability students may not be optimal for foundation students. This in turns suggests the possibility of tailoring teaching to ability, as, for example, is done in mathematics. Whether this means a simple intervention such as slower teaching (and slower more reinforced introduction of new concepts), or different approaches to the teaching, will require considerable further analysis, far beyond the scope of the current analysis. Evident hypotheses for further scrutiny include the possibility that higher-ability students tend to have higher logical reasoning skills. Alternatively, might higher-ability students be any more or less likely to have alternative prior beliefs, compared to lower-ability students?

### Caveats

Given the imperfect nature of school-level interventions, our study naturally comes with multiple caveats. Our strategy was to randomise ordering of teaching between teachers and to have enough teachers in each group as to discount any effect of the individual teacher, cohort or class. That is to say, had we just employed 2 classes we would not have been able to say if any effect was owing to the ordering rather than owing to the teacher. The premise of our design was to assume that with enough different teachers and classes, for every exceptional teacher teaching evolution first, there would be an equally exceptional one teaching genetics first. It remains a possibility that the genetics-first effect was owing to especially good teachers being, by chance, randomised into the genetics first class. Such is the problem of all randomisations. However, we also see the genetics-first effects in both ability partitions (when analysed at the student level). If the core result were a failure of randomisation, it is a failure that has occurred in both independent subgroups. Moreover, we see no evidence for underdispersion, suggesting that teacher effects are not an important issue.

What we can be more confident about is that there exists no bias owing to teachers teaching in their preferred and practiced manner, as teachers didn’t get to select the order that they might teach in (teaching order was handed down from the head of sciences or biology in each school). Those teachers that switched order had no lesser impact on change scores than those that didn’t. Some schools couldn’t, however, be properly randomised, because they were constrained as to order (e.g., they needed more advanced warning to buy in resources). However, there appear to be as many who taught evolution first as taught genetics first, so we don’t expect this to be a major issue. Nonetheless, this constraint means that our trial isn’t of the strict standards of medical randomised control trials in which there are no constraints as to which treatment each recipient is exposed to. Moreover, unlike a medical trial in which there is a treatment and a control, this study permits no control, as it would be unethical to not teach students material they would need in exams. By contrast, as the teachers had to teach the material anyway, we have no evidence of teacher drop out after ordering was chosen (in drug trials, patients can show biased profiles of dropout). There are multiple other issues of sampling (e.g., we invited all schools within a given area, but the responding schools may be a biased subset) that also are less than optimal, but unavoidable as well. We consider these and other details of the implementation of the experimental design in S1 Text.

## Methods

### Ethical considerations and data protection

The project was approved by the Ethics Committee of the Department of Biology and Biochemistry of the University of Bath. Ethical guidelines as prescribed by The British Educational Research Education [63] have been followed. Particular consideration has been taken when working with school students, and approaches that place any undue burden on participants have been avoided. Research through questionnaires and focus groups has taken place within students’ schools and have involved students’ usual science teachers so as to minimise undue intrusion. Additional information including that on consent forms can be found in the supplementary materials (method for obtaining consent to fill questionnaires: S2 Text; questionnaire consent form: S3 Text; method for group consent: S4 Text; focus group permission form: S5 Text; focus group consent form: S6 Text).

### Statement of hypothesis

We consider 3 hypotheses:

*The null hypothesis (HO)*: there is no difference in terms of student response between the genetics-then-evolution and the evolution-then-genetics sequences.

*The positive hypothesis (H+)*: there is an advantage in terms of student response for genetics-then-evolution sequence over evolution-then-genetics sequence.

*The negative hypothesis (H−)*: there is an advantage in terms of student response for evolution-then-genetics over genetics-then-evolution sequence.

Student response we define in 3 dimensions: understanding of evolution, understanding of genetics, and acceptance of evolution. In terms of the student response regarding genetics understanding, we had no prior supposition that order would affect its understanding and so there is no directionality in the test of deviation from null. Effect sizes are expressed as percent difference in increments in scores between treatments. Our design may be considered a revised version of a crossover design [56].

### Statement of intent and design

In trials of this nature, one might suspect that when the test is done, a significant effect of any variety is searched for and a story told about the significant effect. In this context, we wish to state that the study design and intent were lodged with the funders, the Evolution Education Trust in March 2011, the project starting several months after. The project title was “*The teaching of evolution and genetics*: *an investigation into the place of knowledge about genetics in accepting the theory of evolution by natural selection*.*”*

The aim of the project was stipulated thus:

“The structure of the design of the experiment controls for the material presented and allows rigorous evaluation of the hypothesis that ‘front-loading’ the genetics component makes a difference. By comparing the understanding of evolution and genetics before and after the presentation of the packages, we shall be able to determine whether the order of presentation of the genetics-related information has an effect on the probability that a student has improved understanding.”

The full statement of design is available from the funders by contacting the director: Michael Magnay, Church House, Marston St Lawrence, Banbury, Oxon, OX17 2DA, United Kingdom.

#### Modifications after pilot

The experimental design permitted a pilot study and initial consultation with teachers. From this, we chose to a) allow teachers to teach material as they preferred in a manner specific to their exam board (rather than obliging them to teach a preprepared package) and b) to maximize the sample size within the limit of time available. The former option was chosen, as it became clear that teachers would not adopt a significant change to their teaching practice and were the project to realise an order effect (in either direction), then its implementation would be simpler if no change to teaching material was required. Thus, we do not control for material presented.

In addition, we chose to nest the study in a survey of survey of understanding of 14–16-year-old UK school childrens’ understanding and acceptance of evolution prior to any teaching (as here reported). For this, samples sizes in the thousands are the norm, and this informed the choice to maximize. As a priori, the students have to be taught both streams (genetics and evolution) and no information was available to say if either might be better, we presumed no harm. Thus, no power calculation was performed. For considerations of the ideal and constraints on design and mode of randomisation, please consult S1 Text. Randomisation was left to heads of science or biology, and no teacher was permitted to choose their order. Not all schools could alter order (e.g., because they to have to order in supplies for teaching particular lessons), so this project falls short of a full randomisation.

### Student questionnaire

Quantitative data were collected through a student questionnaire to determine acceptance of evolution and understanding of genetics and evolution. This was devised for GCSE-level students (14–16-year-olds) who study evolution and genetics as part of their science GCSE science course. An advantage of analysis of this age group is that order effects may well be most easily detected if there has been little or no priming. While primary school children in the UK are presently expected to be taught basic genetics and evolution on the national curriculum, this is a recent introduction and the cohort we analysed did not have this exposure. Indeed, this academic stage was chosen as it is currently the first, and perhaps only, period at which students have to learn about evolution. This cohort is not self-selecting in the way that a higher academic stage might be. For example, students aged from 16–18 and studying for a Biology A-Level qualification will already have achieved a reasonable standard of academic achievement in science to enrol in this, and presumably have an interest for biology, or would not have chosen to study the subject further. Therefore, in choosing to study GCSE-level students, this research has involved a wide variety of students, in terms of academic ability and interest in evolution and science.

Where possible, the student questionnaire was used at 3 times:

- Pretest—prior to learning both genetics and evolution;
- Post-test—immediately after learning of both topics;
- Retention test—approximately 3 to 6 months after the teaching of the topics (but not coinciding with any revision or examination of topics).

Most schools continued to teach these topics as normal, with existing variation, such as topic order, being comparative between classes without further intervention. However, some schools were also asked to make changes to their normal teaching, such as to change the order in which evolution and genetics were taught, and/or to include an activity which linked the 2 topics. Due to the time constraints of teaching exam specifications and limited flexibility within some school schemes of work, there was no pressure placed on teachers to change their normal teaching sequence or include different resources.

The questionnaire consists of 25 questions: 13 focus on acceptance of evolution (Section A), 6 on genetics knowledge (Section B), and 6 on evolution knowledge (Section C). None of the questions involve extended writing and are all variations of the multiple-choice question. These types of questions were chosen for their practicalities: to aid student completion time, to avoid instances of not being able to understand transcriptions, to allow for quantitative analysis of data, and that this method is commonly used in similar studies (e.g., [49]).

At all stages of the questionnaire development, including a pilot study, evolution and education experts were consulted from the University of Bath along with practising teachers. The questionnaire was designed with time constraints in mind: teachers consulted during its development were insistent that the questionnaire must be short enough so that its completion would not considerably reduce their lesson time. Ten to 15 minutes was considered an appropriate length. The final questionnaires are presented in S7 Text.

#### Evolution acceptance

Section A assesses students’ opinions towards evolution and consists of 13 Likert Scale items. These were based largely on the Measure of Acceptance of the Theory of Evolution (MATE), which was developed to assess biology teachers’ acceptance of evolution [64] and later, undergraduate students’ acceptance of evolution [65]. The original MATE instrument consists of 20 items spread disproportionately across 6 subsections of evolutionary concepts or aspects. It was decided that this was too long for school students. Appropriate questions were chosen based on their relevance to these different aspects of evolution and their accessibility to school-aged students. Given that the MATE has been developed and tested predominately on teachers and undergraduate students (e.g., [49,66]), some modifications to the language used were needed. Where necessary, statements were reworded to make them more understandable. Two items were also based on Lovely and Konderick’s study [67] into undergraduate opinions of evolution. This section was found to be reliable through internal consistency checks (alpha 0.82, G6 0.83).

#### Acceptance categorisation

Student acceptance is categorised based on acceptance scores. Scores for individual items are measured on a scale of 1 to 5, corresponding to “very high acceptance,” “high acceptance,” “undecided,” “low acceptance,” or “very low acceptance” of evolution. Students receive a total score of between 13 and 65 (a higher score represents a higher acceptance of evolution). The acceptance categories referred to here are an amalgamation of the 2 “high” and “low” acceptance categories (“undecided” remains unchanged) as detailed in Table 5. This is based on work by Nadelson and Sinatra [68] to describe MATE categories (after [69]).

**Table 5:** Categorisation of evolution acceptance.

Acceptance Category	Scores
**Low**	13–32
**Undecided**	33–45
**High**	46–65

#### Genetics knowledge

Section B consists of 6 questions which focus on knowledge of genetics. This includes variations on questions from recent GCSE exams, questionnaires used in the Genetics Literacy Assessment Instrument (GLAI) for undergraduates [70], and questions from [71] in their study of school students’ understanding of genetics. Two of these questions involve choosing or ordering key words from lists provided, and one question involves ticking boxes. These types of questions were chosen to gain greater insight into students’ ideas on living organisms and genetics and to add variety to the questionnaire for students.

#### Evolution knowledge

Section C focuses on evolution knowledge and consists of 6 questions. This section includes a variety of different aspects of evolution, including natural selection and geological time. Most of these were variations of questions used by Rutledge and Warden [49] in their research into acceptance and understanding of evolution among high school biology teachers. Additionally, a number of questions were devised with the assistance of evolution experts. Each question was scored equally with a section total out of 6.

#### Data overview

Table 6 gives an overview of responses collected from the student questionnaire. These were collected from a total of 78 classes within 23 different schools between September 2012 and September 2015. The data collection was terminated in order to enable analysis of results within the funding period. In total, 1,886 students completed the questionnaire at least once. Table 7 shows the numbers of students associated with key variables to be investigated, namely ability and topic order. Although 3,572 questionnaires have been received in total, there is a large amount of variation in the number of pre-, post-, and retention tests completed, as can be seen in Table 6. Individual students have been omitted from certain analyses, e.g., those comparing pre- and post-test, where students were absent at the time one of these questionnaires was completed. Data have been used where possible, as there is still value in students’ responses at a single point in time, even if it has not been possible to compare paired data longitudinally. Sample sizes therefore differ considerably for different analyses.

**Table 6:** Overview of student questionnaire data collected.

	Pre	Post	Retention
Number of students	1,716	1,527	329
Number of classes	76	72	18
Number of schools	23	23	8

**Table 7:** Sample sizes for variables of key interest.

Variables	Subsample	Number of students	Number of classes
**Ability**	**Higher**	1456	56
	**Foundation**	430	22
**Topic order**	**Genetics first**	1145	49
	**Evolution first**	741	29

### Focus groups

Focus groups were designed to better understand the responses found in the student questionnaires, i.e., why students were or were not accepting of evolution; how these views related to knowledge of evolution; how these related to knowledge of genetics; and what other factors are important. Seventy-six students were involved in 16 focus groups. These students were from 10 different schools. The largest focus groups contained 7 students and the smallest, 2. All students were from groups identified as “higher-ability,” with most students being from among the top sets in each school. The majority of students were in Years 9, 10, and 11 and studying towards their GCSE examinations. Six students were in Year 12 and studying for A-Level exams. Most focus groups comprised students of the same age and from the same class, however there were 3 groups which contained a mixture of ages and classes.

### Background information

A mixture of state, faith, and independent schools have been involved in this project. All schools are from the South of England and Mid and South Wales. All are English language schools. Schools included students from socially and economically diverse communities, including rural, suburban, and inner city. A number of schools are single-sex. Although data were not collected specifically on student demographics, a wide range of ethnic backgrounds and faiths were represented. Background data on schools have been collected from inspection (OFSTED/ESTYN) reports, school websites, and from meetings with teachers. These can be found in the S1 Data and S1 Text.

#### Statistics

All statistics were conducted in R, via purpose-written Tcl scripts or in JMP. No change in score data was normally distributed (Shapiro test *p* < 10^−16^ in all cases), and we were unable to find a transform to be more normal than the raw data. Thus, we largely employ nonparametric analyses. However, to confirm some results, we additionally performed some parametric tests under the presumption that parametric tests are robust to some degree of deviation from normality (the raw data tends to be slightly skewed). All such analyses come with the inevitable caveats. *Z* refers to Wilcoxon signed-rank test and compares pre- and post-test scores for the paired data (i.e., individual students who took the questionnaire twice). *W* refers to Mann Whitney *U* test and compares different groups of students (i.e., higher- compared to foundation-ability). Internal consistency was evaluated using Cronbach’s alpha. Correlations were nonparametric Spearman correlations.

To determine whether a correlation between *x* and *y* is significantly stronger or weaker than the correlation between *z* and *y*, we performed a nonparametric Monte Carlo simulation. We start by employing only those instances where for any given student, *x*, *y*, and *z* can all be calculated (i.e., students who did all pre- and all post-tests). We then calculated the 2 Spearman correlations and asked about the difference in the Spearman rank coefficient. We then randomised the vector *y*, and considered for each randomised version the correlation between *x* and randomised *y* and *z* and randomized *y*. We again considered the modular difference in Spearman rho value for the correlation of these 2 individually against variable *y* (the mean difference in the simulants is zero). Repeating the simulation 10,000 times, we asked how often the modular difference was as great or greater than that observed in the real data. As we employed modular data, the test is 2-tailed. The type 1 error rate is then given by *p* = (*n* + 1)/(*m* + 1), where *n* is the number of randomizations in which the randomised dispersion is as extreme or more extreme than that observed in the real data and *m* the number of simulations.

Significance is taken at alpha <0.05. The 3 variables (change in evolution acceptance, change in genetics understanding, and change in evolution understanding) we consider to be 3 discrete hypotheses and thus perform no multitest correction to allow for this dimension of multiplicity. However, when stratified by ability, it is reasonable to apply a correction to the 2 partitions, thus if *p* < 0.05/2 is not achieved from the raw statistic, we note that the result is not robust to multitest correction.

We declare that statistical analysis was performed on the data at the end of the trial and the trial was not extended to try and “find a result.”

Item nonresponse levels were low. We considered alternative means to handle nonresponse, but as the numbers are so low, they make no difference to results (S1 Text). All raw data can be found in S1 and S2 Data. S1 Data present the information at a class level. S2 Data presents the score data against the individual (anonymised) student. Note that the class id is the first digits of the student id.

### Consort checklist

The consort guidelines provide specification of desirable statements regarding the design and implementation of a randomised control trial. We provide this checklist as S8 Text. The page numbers referred to specify the pages in the draft manuscript available at S9 Text.

## Supporting information

S1 Text Additional discussion and information. (DOCX)

S2 Text Method for questionnaire consent. (DOCX)

S3 Text Questionnaire consent form. (DOCX)

S4 Text Focus group consent method. (DOCX)

S5 Text Focus group permission form. (DOCX)

S6 Text Focus group consent form. (DOCX)

S7 Text Questionnaire. (PDF)

S8 Text CONSORT checklist. (DOC)

S9 Text Manuscript to cross reference page numbers in S8. (PDF)

S1 Data Class level data. (XLSX)

S2 Data Pupil level data. (XLSX)

## Abbreviations

GCSE: General Certificate of Education
GLAI: Genetics Literacy Assessment Instrument
GM: genetically modified
MATE: Measure of Acceptance of the Theory of Evolution
