# Supplementary material for: Teaching genetics prior to teaching evolution improves evolution understanding but not acceptance
Source: PLoS Biol. 2017 May 23;15(5):e2002255. doi: 10.1371/journal.pbio.2002255 (PMC5441579; doi:10.1371/journal.pbio.2002255)
Supplement: S1 Text — (DOCX) [file pbio.2002255.s001.docx]

**Additional Information**

**Randomized control trial**

Randomizing classes to ‘treatment’ and ‘control’ groups: this is not particularly accurate as we don’t have a ‘control’ or ‘treatment’ *per se*. Before our ‘intervention’, schools taught evolution and genetics in different orders:

- some schools taught genetics first then evolution immediately after
- some schools taught evolution first then genetics immediately after
- a few schools taught genetics first then evolution later (this varied from a few months to over a year! We looked at the impact of this but found nothing of significance: numbers of students within these schools likely accounted for this)
- schools may have taught evolution first then genetics months later, but this was never stated by the schools involved in this study
- a few schools taught a mixture of the above topic orders (typically this was to rotate classes so that enough equipment for practicals would be available when needed)

These decisions on topic order were generally made at the whole-school (or science department) level, rather than left to individual teachers, and often reflected the particular syllabus followed (i.e. AQA specification places genetics immediately before evolution whereas Edexcel places evolution before genetics. OCR specifications typically have gaps between genetics and evolution).

Therefore, there was no particular ‘control’ to begin with (i.e. it was not the case that all schools taught genetics first and that this was the default ‘control’) and there was no trialing of a new ‘treatment’ (i.e. a complete change to what was usually taught). We were looking at two topics that would be taught anyway: we weren’t introducing anything new.

**Methodology**

In an ideal scenario, the methodology for this is as follows:

- Have at least two comparable (e.g. top two) classes within each school
- Half of the classes involved in each participating school would continue to learn in the ‘normal’ topic order; half would learn in the opposite order.
- This was based on the assumption that, prior to involvement in this study, roughly half of all schools involved would normally teach evolution first, and that roughly half would teach genetics first. These proportions should remain roughly the same (or indeed, become closer to 50:50) during participation.
- The head of science/head of biology would be asked to randomly pick which class(es) would learn in which topic order.
- It would also be also left to the head of science/biology to decide the extent to which classroom teachers were aware of the project design and reasons for changing the topic order. Teachers were not ‘blind’ to the study design; however, students were.

So as not to deter participation, it was emphasized that it was the school/head of science’s decision as to whether topic order could be changed and which classes should change topic order. This may have led to less randomization than is ideal, but it must be stated that within an authentic classroom setting, sometimes it was not practical, due to the many pressures schools face, for the methodology to be followed exactly. In this structure, the demographics of genetics first and evolution first treatments should be the same as the schools did both.

Problems that led to deviation from the ideal scenario methodology:

- Not all schools were willing to involve more than one class
  - This was most likely to be the case in small schools or schools where only one particular teacher was interested in involvement with the study
- Similarly, some schools were happy to involve multiple classes, but these were sometimes an odd number
  - Decisions about how many classes should swap order were left to head of science/biology
- Not all schools were in a position where they thought they could change the order of topics. This was usually due to perceived time constraints (sometimes justified, e.g. if teachers were ready to start teaching on topic but were not yet prepared to teach another) and opinion that senior management would not allow any changes to normal teaching order
- Not all schools who agreed to participate returned questionnaires
  - A large number of questionnaires were never returned or were returned too late to be included
  - Reasons for this included changes in staffing (affected at least two schools who had requested large numbers of questionnaires and agreed to changing the topic order). Other than this the reasons are largely unknown but involvement may have been considered too time consuming (many schools admitted to being under huge time pressures and perhaps any changes were just too great) or there may have been resistance at the managerial or classroom level.
- Not all schools changed the topic order as prearranged
  - Reasons for this were unclear but may have been due to poor communication between head of science/biology and class teachers, changes in staffing, and/or resilience of classroom teachers to teacher differently to normally
- Generally: the quality of descriptive data received from school was variable.
  - E.g. Information from initial meetings with heads of department, etc. didn’t always tally with post-questionnaire information given by class teachers. This might represent real differences, e.g. over time or between teachers (lack of knowledge of head of department about what was actually happening in all classes?) or may have been given in error.

We did not control for teacher effects. There are numerous reasons for this including a lack of widely accepted way of doing so and disagreement of teacher effects. Within this study there was variation between the potential impact individual teachers could have had:

- Some teachers taught more than one class
  - Some were able to teach their different classes using different topic orders
  - Some were unable to do this
- Some classes had more than one teacher
  - Shared classes (e.g. teachers who work part time)
  - Staffing changes within the year
  - Temporary trainee teachers, supply teachers, etc.

Although it could be argued that these are not ideal settings, again, we reiterate that this study took place in an authentic rather than controlled classroom setting. We want the findings to be applicable to a wide range of authentic classroom settings, and these situations are what happen regularly within schools. Therefore this should not be viewed negatively.

It should also be noted that it was not usually an individual teacher’s choice as to whether they taught a particular topic order or not: this was usually a decision made at a higher-level, e.g. by the head of science or head of biology, and these were asked to make such decisions randomly (although this may not have always been practical, e.g. if a particular teacher was ahead or behind in their current teaching, this may have meant they were or were not in a position to change topic order). Therefore any potential impacts of only ‘keen’ or ‘excellent’ teachers being willing to swap topic order or deviate from their normal teacher should be non-existent.

(Note: some class teachers appeared to work somewhat independently compared to the whole school, so what may have been accurate for a school as a whole may not have been correct for every teacher, e.g. topic order. Some schools were more stringent about this than others. It should also be mentioned that as genetics and particularly evolution tend to appear last on exam board specifications, teachers may have had to jump to perceived ‘important’ topics that were likely to be examined and gloss over ‘lesser’ areas. Although I asked for individual information from each classroom teacher through a short question sheet, some heads of science/biology completed them for all teachers, hence removing any individual input from class teachers.)

**Representative sampling and potential bias**

All schools within the South of England and South and Mid Wales invited to participate.

An alternative approach may have been to target particular *types* of schools. However, what would this have been based on?

- School type?
  - All school types were invited to participate in this study
  - Representation in each group is *roughly* representative of the proportions of school types nationally. However, in reality, there are large regional differences (e.g. grammar schools and independent schools are more common in certain counties)
- Location? I.e. better national representation?
  - Could be harder to recruit schools:
    - Meetings with teachers
    - Encouraging school involvement with University activities by means as a ‘thank you’
- Socio-economic factors?
- Exam board?
  - Not all schools make this information available (as can be seen from the data, particular exam boards were more common than other, although this is true for most subjects – one exam board is often more dominant than others)
  - Some schools use more than one exam board
- Topic order?
  - Almost impossible to discover without discussion with heads of department/class teachers (i.e. most schools don’t openly publish this type of detail on websites, etc. and this information is usually only known to those within the science department)

Main downfall of adopting this approach (based on any of the above criteria):

- Sample size likely to be considerably smaller

**Other**

It should be reiterated that questionnaires were all conducted in authentic rather than controlled classroom environments.

No attempt to standardize the ways in which evolution or genetics were taught. Each exam board specification is different with some placing more emphasis on genetics and/or evolution than other (i.e. IGCSE courses typically give evolution less coverage).

Education research differs from areas such as clinical or laboratory research. It is less focused on seeking irrefutable ‘facts’ and instead offers to seek new insights, acknowledging potential subjectivity and threats to validity.

This study may use an ‘imperfect’ sample but given the large number of students and the fact that we are not controlling for the many variables… representative of school students within a wide variety of school types, etc.

**Item non-response**

Item non-response refers to instances of respondents not answering particular items within a research instrument. It can have a significant impact on the quality of data. Missing data can lead to misinterpretations of analyses of individual questions. For example, if a question appears to have low item difficulty but high non-response, are only the students who understand that question and know the answer responding? Item non-response can also be problematic in analysing data. For example, if a student answers 12 of the 14 evolution acceptance items in a highly accepting of evolution manner but gives no response to two of the questions, should these be scored as zero, hence lowering their overall acceptance score, or should allowances for missing data be made? Missing data require careful consideration.

Missing data due to student absence (i.e. where an individual student completed at least one questionnaire, but was not present when their class completed the questionnaire at a different point in time) are not included in comparative analyses, e.g. those using paired data.

The reasons for item non-response are clearly important, but there is often little or no indication as to why individuals have failed to respond to all items. Potential reasons include accidental skipping of questions, lack of interest or attention, disturbances or distractions, time constraints, not wanting to answer a question (e.g. for personal/faith reasons), not understanding a question or certain words within an item, not knowing how to answer a question, and/or not knowing the answer to a question. Some of these possibilities are external factors and not likely to be due to the design of the questionnaire (although it could be argued that the layout and number of items may impact on these). Others are more directly linked to the research instrument.

Instances of item non-response for the student questionnaire are shown in Table 1. These are displayed as a proportion of students present when the questionnaire was completed at each stage. There were a number of ways in which item non-response occurred:

- A student did not attempt a question – there was no answer;
- A question was answered in an ambiguous way (e.g. two answers circled when only one was required);
- A question was answered in an incorrect way that suggested a student did not understand the instruction for the question (e.g. using the same word as an answer twice in a question that required individual words to be used once only).
- A question was answered in a bizarre or crude manner.

These have not been differentiated between in this analysis: all are recorded as occurrences of item non-response.

| **Question** | **Pre** | **Post** | **Retention** | **All (mean)** |
| --- | --- | --- | --- | --- |
| **Q1** | 0.23 | 0.39 | 0.30 | 0.31 |
| **Q2** | 0.87 | 1.11 | 0.30 | 0.92 |
| **Q3** | 1.16 | 1.11 | 0.30 | 1.06 |
| **Q4** | 0.82 | 0.65 | 0.00 | 0.67 |
| **Q5** | 0.70 | 0.85 | 0.61 | 0.76 |
| **Q6** | 1.22 | 1.18 | 0.30 | 1.12 |
| **Q7** | 0.87 | 0.98 | 0.30 | 0.87 |
| **Q8** | 0.99 | 1.24 | 0.00 | 1.01 |
| **Q9** | 0.64 | 0.98 | 1.22 | 0.84 |
| **Q10** | 2.33 | 2.42 | 1.52 | 2.29 |
| **Q11** | 1.16 | 1.11 | 0.30 | 1.06 |
| **Q12** | 0.82 | 1.11 | 0.30 | 0.90 |
| **Q13** | 0.76 | 2.55 | 1.82 | 1.62 |
| **Q14** | 0.82 | 3.08 | 1.82 | 1.88 |
| **Q15** | 4.66 | 5.89 | 1.52 | 4.90 |
| **Q16a** | 3.55 | 5.44 | 1.22 | 4.14 |
| **Q16b** | 5.53 | 6.88 | 1.82 | 5.77 |
| **Q16c** | 5.53 | 6.94 | 1.82 | 5.79 |
| **Q16d** | 5.36 | 7.66 | 2.43 | 6.07 |
| **Q16e** | 5.01 | 6.88 | 3.65 | 5.68 |
| **Q16f** | 8.15 | 10.81 | 9.42 | 9.40 |
| **Q16g** | 6.29 | 10.02 | 4.56 | 7.72 |
| **Q16h** | 1.51 | 4.98 | 0.61 | 2.91 |
| **Q16i** | 5.30 | 10.15 | 6.69 | 7.50 |
| **Q16j** | 15.43 | 23.05 | 24.32 | 19.51 |
| **Q16k** | 15.26 | 23.05 | 24.62 | 19.45 |
| **Question (cont.)** | **Pre** | **Post** | **Retention** | **All (mean)** |
| **Q16l** | 15.49 | 23.05 | 24.92 | 19.59 |
| **Q16m** | 4.19 | 6.75 | 2.43 | 5.12 |
| **Q16n** | 4.66 | 7.07 | 2.43 | 5.49 |
| **Q16o** | 6.17 | 7.27 | 2.13 | 6.27 |
| **Q16p** | 7.57 | 9.89 | 5.17 | 8.34 |
| **Q16q** | 6.12 | 9.82 | 6.38 | 7.72 |
| **Q16r** | 7.40 | 10.87 | 10.03 | 9.12 |
| **Q16s** | 3.84 | 5.83 | 0.91 | 4.42 |
| **Q16t** | 5.18 | 6.75 | 0.91 | 5.46 |
| **Q16u** | 6.64 | 7.86 | 2.13 | 6.75 |
| **Q16v** | 9.26 | 10.48 | 6.69 | 9.54 |
| **Q16w** | 10.02 | 12.57 | 7.60 | 10.89 |
| **Q16x** | 10.95 | 13.88 | 9.12 | 12.03 |
| **Q17a** | 12.52 | 11.92 | 6.38 | 11.70 |
| **Q17b** | 15.03 | 11.53 | 4.86 | 12.59 |
| **Q17c** | 12.00 | 12.12 | 5.47 | 11.45 |
| **Q17d** | 15.84 | 12.70 | 4.56 | 13.46 |
| **Q17e** | 14.04 | 12.25 | 3.65 | 12.31 |
| **Q17f** | 16.66 | 14.15 | 7.29 | 14.72 |
| **Q18** | 8.21 | 10.02 | 5.78 | 8.76 |
| **Q19** | 6.76 | 8.32 | 4.56 | 7.22 |
| **Q20** | 6.35 | 9.04 | 5.78 | 7.44 |
| **Q21** | 6.93 | 8.97 | 7.60 | 7.86 |
| **Q22** | 12.29 | 12.25 | 9.12 | 11.98 |
| **Q23** | 9.03 | 11.13 | 7.60 | 9.80 |
| **Q24** | 10.66 | 11.33 | 7.60 | 10.66 |
| **Q25** | 12.58 | 11.92 | 8.21 | 11.89 |
| **Q26** | 12.99 | 12.05 | 9.42 | 12.26 |

**Table 1** Percentage item non-response (Pre N=1886; post N=1886; retention N=434; overall N=4206).

Item response varies between items. For this reason, findings throughout this research may be presented as a proportion of those who did answer a particular question, where appropriate, and sample size will vary.

Overall, item non-response was higher in Sections B and C than in Section A. Question 16 had the highest levels of non-response. It is thought this may be due to students failing to annotate a question when they thought ‘no’ was the correct answer. Missing data towards the end of the questionnaires may be due to students running out of time; however, comparisons with item difficulty suggest these students found questions on the last page the hardest. It is therefore difficult to make any conclusions about the reasons for item non-response.

Six different strategies were considered when dealing with item non-response. These are displayed in Table 2. Due to the relatively low instances of missing data, none of these approaches appear to make any difference to analyses such as reliability. It was therefore decided to use different strategies within the different types of sections: **missing data were awarded a score of ‘0’ in knowledge-based sections and non-response evolution acceptance items were scored as ‘3’. Students who answered less than half of the questions within the evolution acceptance section were removed from this section**.

| **Strategy** | **Advantage** | **Disadvantage** |
| --- | --- | --- |
| 1. Give score of ‘0’ (zero) for every instance of non-response. | • Includes all students. • No assumptions about what score a student might have had. | • May give students an unjustly low score, i.e. if the question were omitted by accident. • Interferes with evolution acceptance scoring systems, i.e. possible to have a score lower than 14. |
| 2. Remove all students who have missed items. | • No assumptions about why students didn’t answer questions. • No assumptions about what score a student might have been had. • No awarding of unjustly low or high marks. | • Lowers sample size. • May ignore important responses. |
| 3. Delete students who have a high percentage of missing data, e.g. over 50% item non-response. | • Removal of the least reliable students. | • Still need strategy to deal with remaining students' non-response items. |
| 4. Replace omitted item with the overall mean for that item. | • Includes all students • Less assumptions about individual scores. • Does not interfere with evolution acceptance scoring system, i.e. no score can be lower than 14. | • Many ways of doing this, e.g. based on overall responses for a particular class or ability, non of which may give a true reflection of the score an individual would have had. |
| 5. Scale up an individual's marks based on their other answers. | • Includes all students. • Students receive marks consistent with their responses. • Does not interfere with evolution acceptance scoring system, i.e. no score can be lower than 14. | • Assumptions about how questions would have been answered, i.e. that omitted questions would have been answered in a similar way. |
| 6. Replace missing data with an 'undecided' score. | • Includes all students. • Does not interfere with evolution acceptance scoring system, i.e. no score can be lower than 14. | • Only applicable to evolution acceptance items. • Still need strategy to deal with knowledge non-response items. • Assumptions about how questions would have been answered, i.e. that items were omitted due to student being undecided. |

**Table 2** Strategies considered for dealing with non-response items.
