# Supplementary material for: Teaching genetics prior to teaching evolution improves evolution understanding but not acceptance
Source: PLoS Biol. 2017 May 23;15(5):e2002255. doi: 10.1371/journal.pbio.2002255 (PMC5441579; doi:10.1371/journal.pbio.2002255)
Supplement: S2 Text — (DOCX) [file pbio.2002255.s002.docx]

**Student questionnaires**

Consent forms for the student questionnaire have been produced and distributed to teachers. They consist of information for parents or guardians and are to be completed and returned to class teachers if students are **not** permitted to be involved in the research project. These are shown and discussed with teachers at initial meetings. The majority of teachers, in agreement with their Head of Department and/or Head Teacher, did not use these forms as they felt the questionnaires were part of their students’ regular science education, and as they took place in usual lesson time, consent was not needed. However, students were made aware that it was their choice to complete the questionnaire and were able to withdraw from the study should they wish.
