# Supplementary material for: Teaching genetics prior to teaching evolution improves evolution understanding but not acceptance
Source: PLoS Biol. 2017 May 23;15(5):e2002255. doi: 10.1371/journal.pbio.2002255 (PMC5441579; doi:10.1371/journal.pbio.2002255)
Supplement: S3 Text — (DOCX) [file pbio.2002255.s003.docx]

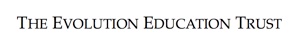

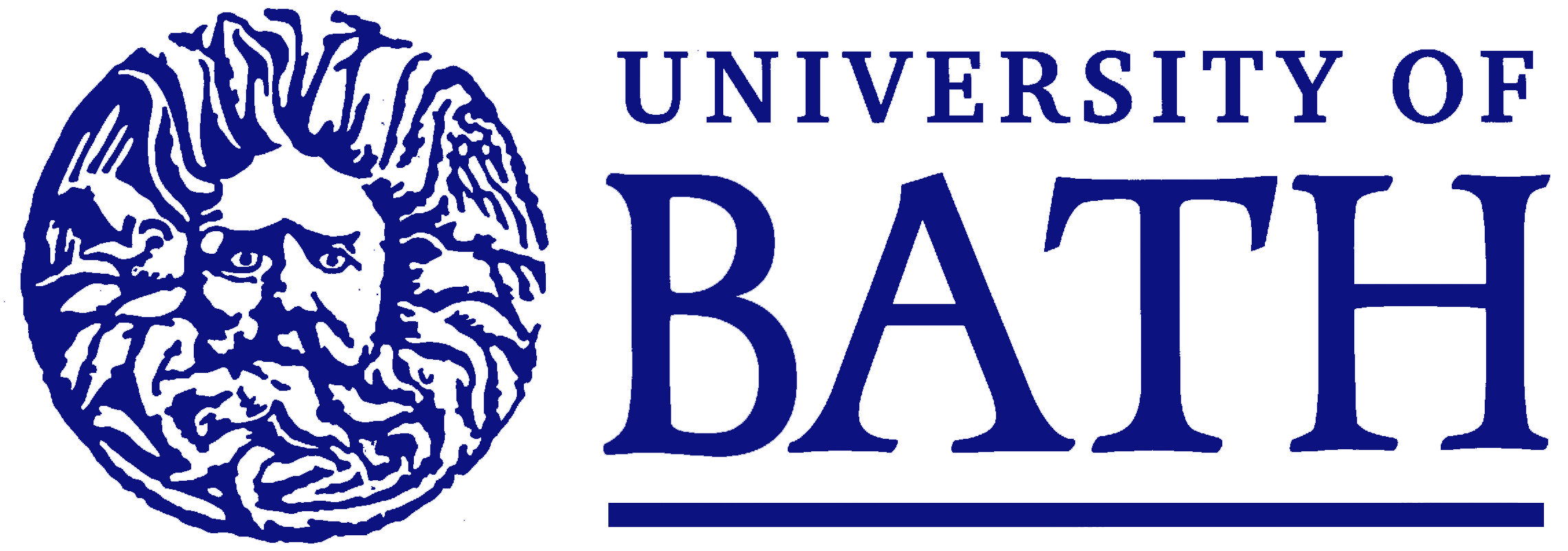

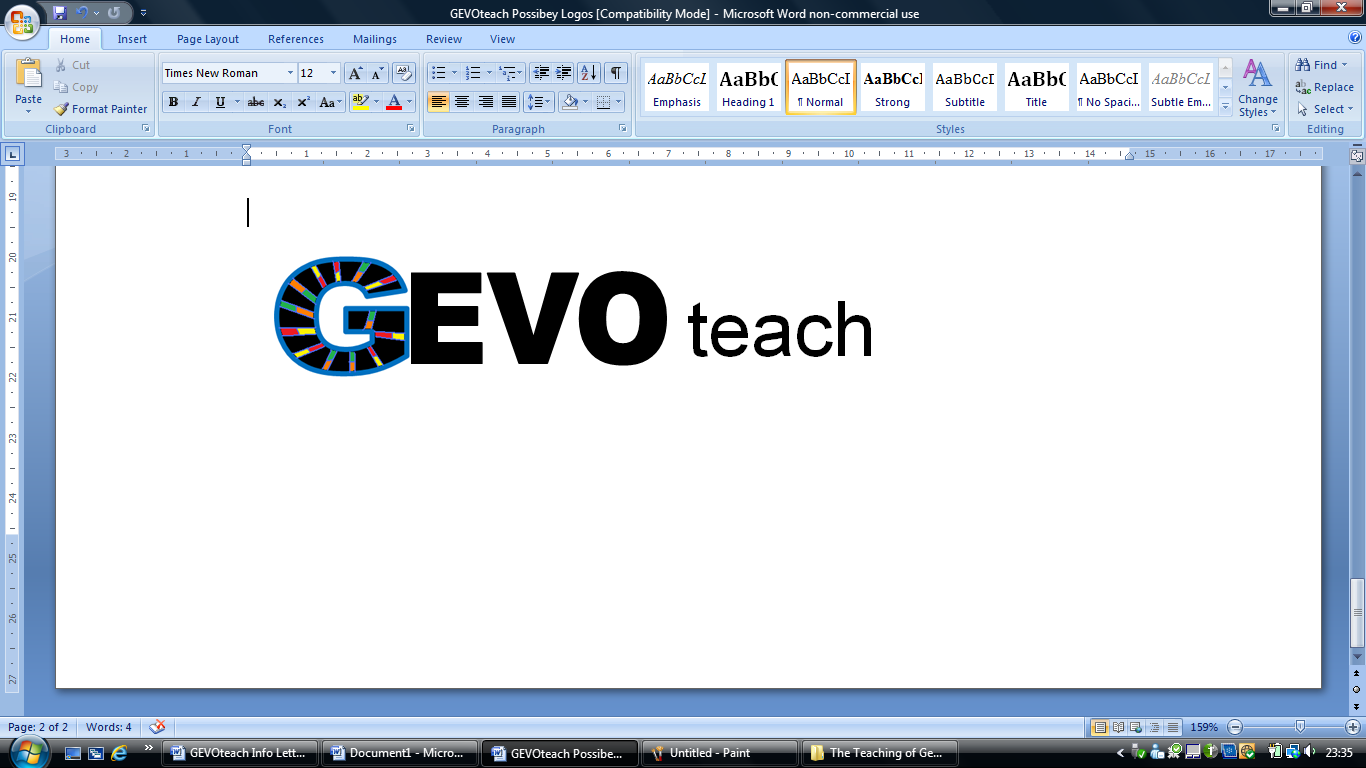

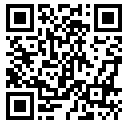


[Date]

Dear Parent/Guardian,

I am pleased to inform you that your son/daughter’s class will be working alongside the University of Bath and the Evolution Education Trust on the GEVOteach Project. This exciting new research initiative aims to improve evolution and genetics education in secondary schools.

As part of our research, pupils will be asked to answer a short questionnaire about their opinions and knowledge of evolution and genetics. This will take place in their normal science classroom. All data will be kept strictly confidential: no responses will be linked to pupil names and individual data will only be seen by members of the GEVOteach research team.

More information about the research can be found on the project website (<http://go.bath.ac.uk/GEVOteach>). If you have any queries, please speak to your child’s biology teacher or contact me directly by phone or email.

If you are **unwilling** for your son/daughter to be involved in this research, please complete the below form and return it to their biology teacher. (Please note, you do **not** need to return this form if you are happy for your son/daughter to participate in this research.)

I thank you in advance for your support.

Yours faithfully,

*R Mead*

Rebecca Mead

Postgraduate Researcher

Department of Biology and Biochemistry

University of Bath

Bath

BA2 7AY

Tel: 01225 385902

Email: [r.mead@bath.ac.uk](mailto:r.mead@bath.ac.uk)

Website: <http://go.bath.ac.uk/GEVOteach>

twitter: <http://twitter.com/GEVOteach>


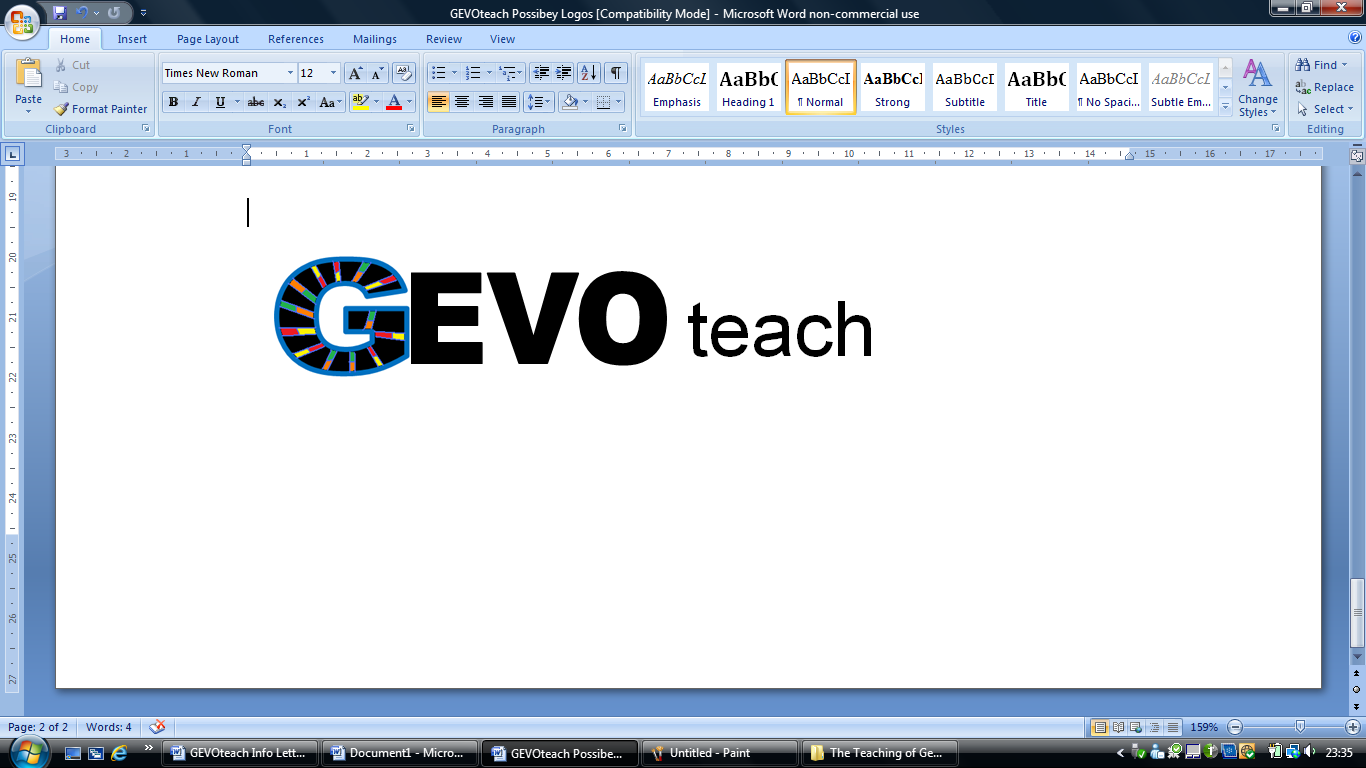


***School Name***

Pupil Name _________________________ Form/Science Class ________________

I **do not** give consent for my son/daughter to complete any questionnaires in connection with the GEVOteach Project

Parent/Guardian Name _________________ Signature ________________ Date ________
