# Supplementary material for: Teaching genetics prior to teaching evolution improves evolution understanding but not acceptance
Source: PLoS Biol. 2017 May 23;15(5):e2002255. doi: 10.1371/journal.pbio.2002255 (PMC5441579; doi:10.1371/journal.pbio.2002255)
Supplement: S4 Text — (DOCX) [file pbio.2002255.s004.docx]

**Student focus groups**

Particular care was taken regarding permission and consent for participation in the student focus groups. Teachers were provided with information about what the focus groups would involve and were tasked with selecting suitable students (who themselves had to be willing to participate) and arranging an appropriate venue and supervision for the focus group. Students who were willing to participate were provided with permission forms, to be completed by their parents or guardians. These contained information about the purpose of the research project, the format of the focus group, and confidentiality. If permission was given, these forms had to be signed and returned to the students’ teacher, before the day of the focus group.

Immediately before each focus group, the format of the session was discussed with students. The use of a dictaphone and confidentiality were discussed, and students were asked to respect the views of other participants. Students were informed that they were free to leave the group at any point and that they did not have to answer any questions. Students also had the opportunity to ask any questions and were given project information sheets to keep, should they later wish to contact the researchers. Students then completed consent forms prior to the focus group commencing. An adult from the students’ school was present throughout every focus group.

All permission and consent forms have been kept securely and no personal information has been computerised (students are identified by codes). Recordings of focus groups have been password protected. An external transcription company was used to transcribe recordings. Transcriptionists had signed legally binding confidentiality agreements and all audio files and transcripts held by the company were deleted after they had been delivered.
