# Supplementary material for: Teaching genetics prior to teaching evolution improves evolution understanding but not acceptance
Source: PLoS Biol. 2017 May 23;15(5):e2002255. doi: 10.1371/journal.pbio.2002255 (PMC5441579; doi:10.1371/journal.pbio.2002255)
Supplement: S5 Text — (DOCX) [file pbio.2002255.s005.docx]

[Date]

Dear Parent/Guardian,

I am pleased to inform you that your son/daughter has been invited to participate in a focus group for the University of Bath’s GEVOteach Project. Please read this information sheet and complete the attached permission form if you are happy for your son/daughter to participate.

**What the project is about**

The GEVOteach Project is an exciting new research initiative which aims to improve the teaching of genetics and evolution in secondary schools. This study is under the supervision of Professor Laurence D Hurst from the Department of Biology and Biochemistry at the University of Bath. This research is funded by the Evolution Education Trust. More information about this research can be found on the project website (<http://go.bath.ac.uk/GEVOteach>).

**What the focus group is about**

The purpose of this focus group is to explore young peoples’ views and knowledge of evolution and genetics. These are topics that your son/daughter has recently studied as part of their GCSE science course.

**What your son/daughter will be asked to do**

Your son/daughter will be asked to participate in a focus group. This will take the form of a relatively informal small discussion group with approximately five other pupils from their science class. Each pupil will be asked opinion-based questions related to what they have learnt in their science classes. They will also be invited to respond to comments other members of the group make. The session will last no longer than one hour and will take place during your son/daughter’s normal science lesson, in their school, on [date]. A member of school staff will remain within sight of the group for the duration of the session.

**Benefits and risks**

Findings from this research may help improve how evolution and genetics are taught in schools, which could benefit current and future school pupils. This study may prove an interesting experience for pupils to gain insight into biology and education research and will also be an opportunity for pupils to reflect on their learning. No risks greater than those experienced in ordinary conversation are anticipated. Everyone involved in the focus group will be asked to respect the privacy of the other group members.

*PTO*

**Taking part is entirely voluntary**

Your decision as to whether or not your son/daughter can participate in this focus group will have no impact on their school education or on any current or future relationship with the University of Bath. Your son/daughter will also be asked to sign a consent form prior to participating in the focus group and will be free to withdraw from the study at any time.

**All responses are confidential**

The focus group will be recorded using a dictaphone so that it can be transcribed later (by myself); no one else will hear this and the recording will be destroyed once transcribed. Your son/daughter will never be identified by name in the transcription of the focus group or in any reports published as part of this research. All data will be kept strictly confidential: all materials will be stored in a secure location and individual data will only be seen by members of the GEVOteach research team.

If you have any queries, please speak to your son/daughter’s biology teacher or contact me directly by phone or email.

If you are **willing** for your son/daughter to be involved in this research, please complete the attached form and return it to their biology teacher **before [date]**.

I thank you in advance for your support.

Yours faithfully,

*R Mead*

Rebecca Mead

GEVOteach Project Postgraduate Researcher

Department of Biology and Biochemistry

University of Bath

BA2 7AY

tel: 01225 385902 website: <http://go.bath.ac.uk/GEVOteach>

email: [r.mead@bath.ac.uk](mailto:r.mead@bath.ac.uk) twitter: <http://twitter.com/GEVOteach>

**Focus Group Permission Form**

**Statement of consent:**

I have read and understood the attached information.

By signing below, I give permission for my son/daughter to participate in a focus group for the GEVOteach Project.

Pupil Name _________________________________________________________

Form/Science Class __________________________________________________

Parent/Guardian Name ________________________________________________

Signature __________________________________________________________

Date ______________________________________________________________

~ Thank you ~

All responses will be kept strictly confidential
