# Supplementary material for: Teaching genetics prior to teaching evolution improves evolution understanding but not acceptance
Source: PLoS Biol. 2017 May 23;15(5):e2002255. doi: 10.1371/journal.pbio.2002255 (PMC5441579; doi:10.1371/journal.pbio.2002255)
Supplement: S6 Text — (DOCX) [file pbio.2002255.s006.docx]

**GEVOteach Focus Group Information Sheet**

This focus group is part of a PhD student project which is looking at young peoples’ views and knowledge of evolution and genetics.

- The focus group should last about 60 minutes.
- The focus group will be recorded, and then transcribed so that we have a record of the discussion.
- When we transcribe the focus group we will change your name and any references that you make to specific people and places to ensure that no-one can be identified from our data.
- Nothing you say in this focus group will be heard by anyone else in your school or at the University. All responses are confidential and we ask that all focus group participants respect the privacy and views of other group members.
- If you agree to take part in this focus group, but feel at any stage that you would like to stop, you are free to do so at any time, without giving any reason.

If you (or anyone else) have any questions about this project, then feel free to contact us:

Rebecca Mead

GEVOteach Project Postgraduate Researcher

Department of Biology and Biochemistry

University of Bath

BA2 7AY

tel: 01225 385902 website: <http://go.bath.ac.uk/GEVOteach>

email: [r.mead@bath.ac.uk](mailto:r.mead@bath.ac.uk) twitter: <http://twitter.com/GEVOteach>

**(Participant keeps this section)**

**Focus Group Consent Form**

**Statement of consent:**

I have read and understood the information sheet for the GEVOteach research project and I have had the opportunity to ask any questions I have about the research.

I agree to participate in a focus group for the GEVOtech project that will be audio-recorded and transcribed. Transcripts will be used only for the purposes of this research project, for as long as this research is being undertaken.

The researchers will not use my personal data for any other purpose or disclose it to any third parties. The information I provide, in the form of my comments, will be anonymised (e.g. my name will be removed and replaced with a number). I agree to parts of what I say being used anomalously by the researchers in publications and presentations.

Participant Name _________________________________________________________

Participant Signature ______________________________________________________

Researcher Name _________________________________________________________

Researcher Signature ______________________________________________________

Date ___________________________________________________________________

**(Two copies required: one to be kept by the interviewee, one to be kept by the researcher)**

~ Thank you ~

All responses will be kept strictly confidential

**Focus Group Consent Form**

**Statement of consent:**

I have read and understood the information sheet for the GEVOteach research project and I have had the opportunity to ask any questions I have about the research.

I agree to participate in a focus group for the GEVOtech project that will be audio-recorded and transcribed. Transcripts will be used only for the purposes of this research project, for as long as this research is being undertaken.

The researchers will not use my personal data for any other purpose or disclose it to any third parties. The information I provide, in the form of my comments, will be anonymised (e.g. my name will be removed and replaced with a number). I agree to parts of what I say being used anomalously by the researchers in publications and presentations.

Participant Name _________________________________________________________

Participant Signature ______________________________________________________

Researcher Name _________________________________________________________

Researcher Signature ______________________________________________________

Date ___________________________________________________________________

**(Two copies required: one to be kept by the interviewee, one to be kept by the researcher)**

~ Thank you ~

All responses will be kept strictly confidential
