# Supplementary material for: Teaching genetics prior to teaching evolution improves evolution understanding but not acceptance
Source: PLoS Biol. 2017 May 23;15(5):e2002255. doi: 10.1371/journal.pbio.2002255 (PMC5441579; doi:10.1371/journal.pbio.2002255)
Supplement: S7 Text — (PDF) [file pbio.2002255.s007.pdf]

Section A. *Please circle ‘strongly agree’, ‘agree’, ‘undecided’, ‘disagree’, or ‘strongly disagree’ to indicate your opinion of each of the following statements.*

1. Plants and animals that are alive today are the result of evolutionary processes that have occurred over millions of years.
   Strongly Agree Agree Undecided Disagree Strongly Disagree

2. The theory of evolution is based on guesswork and not valid scientific observation and evidence.
   Strongly Agree Agree Undecided Disagree Strongly Disagree

3. Modern humans are the product of evolutionary processes that have occurred over millions of years.
   Strongly Agree Agree Undecided Disagree Strongly Disagree

4. There are large amounts of factual, historical, and laboratory data that support evolutionary theory.
   Strongly Agree Agree Undecided Disagree Strongly Disagree

5. Most scientists accept evolutionary theory to be correct.
   Strongly Agree Agree Undecided Disagree Strongly Disagree

6. It is not possible to scientifically disprove the theory of evolution.
   Strongly Agree Agree Undecided Disagree Strongly Disagree

7. The first humans lived at the same time as dinosaurs.
   Strongly Agree Agree Undecided Disagree Strongly Disagree

8. The theory of evolution cannot be correct since it disagrees with religious accounts of creation.
   Strongly Agree Agree Undecided Disagree Strongly Disagree

9. The age of the earth is less than 20,000 years.
   Strongly Agree Agree Undecided Disagree Strongly Disagree

10. Evolutionary theory generates testable predictions with respect to the characteristics of life.
    Strongly Agree Agree Undecided Disagree Strongly Disagree

11. Much of the scientific community doubts if evolution occurs.
    Strongly Agree Agree Undecided Disagree Strongly Disagree

12. Humans exist today in the same form in which they always have.
    Strongly Agree Agree Undecided Disagree Strongly Disagree

13. Dinosaurs and humans have never existed at the same time.

Strongly Agree Agree Undecided Disagree Strongly Disagree

14. The age of the earth is approximately 4-5 billion years.

Strongly Agree Agree Undecided Disagree Strongly Disagree

Section B.

15. Place the following items in order of size, starting with the largest first:

cell, chromosome, gene, organism, nucleus

Largest:

Smallest:

16. Complete the following table by placing a tick (to give the answer 'yes'), cross (to give the answer 'no') or question mark (to give the answer 'unsure') in each box.

	**Are they living organisms?**	**Are they animals?**	**Are they made up of cells?**	**Do they contain genetic information?**
Humans				
Giraffes				
Moths				
Oak Tree				
Bacteria				
Virus				

17. Complete the following table by using the words in the box below. Each word may be used once, more than once, or not at all. Only one word is needed for each definition.

environmental		*genetic*	*chromosome*	*sexual*
	DNA	*alleles*	*asexual*	
clone	*variation*	*gamete*	*nucleus*	*mutation*

**Word**	**Definition**
	Different forms of genes
	A change to the DNA sequence
	An individual that is genetically identical to its parents
	A sex cell
	Reproduction which needs only one parent
	Variation which is due to inheritance

*Please circle the correct answer for the following questions. Each question only has **one** correct answer*

18. Muscle cells, nerve cells, and skin cells have different functions because each type of cell
    A. Contains different kinds of genes.
    B. Is located in different areas of the body.
    C. Activates different genes.
    D. Contains different numbers of chromosomes.

19. How do plants reproduce?
    A. Sexual reproduction.
    B. Asexual reproduction.
    C. Both sexual and asexual reproduction.
    D. Plants do not reproduce.

20. Transferring genes from one organism to another is called
    A. Cloning.
    B. Evolution.
    C. Genetic engineering.
    D. Tissue culture.

Section C. *Please circle the correct answer for each question. Each question only has* ***one*** *correct answer.*

21. Which of the following phrases best describes evolution?
    A. The development of man from monkey-like ancestors.
    B. The change of simple to complex organisms.
    C. The development of characteristics in response to need.
    D. Genetic changes in populations through time.

22. Which of the following is not a part of the theory of evolution by natural selection?
    A. Individuals of the same species show variation.
    B. Organisms tend to produce more offspring than survive to adulthood.
    C. Modifications an organism acquires during its lifetime can be passed to its offspring.
    D. Some characteristics can be inherited.

23. Which of the following statements is most acceptable from an evolutionary point of view?
    A. Apes and humans have evolved from a common ancestor.
    B. Humans have evolved from apes.
    C. Certain apes have gradually evolved into humans.
    D. Apes and humans are related.

24. Scientists surveyed the number of light and dark coloured moths in woods where trees had either light or dark (polluted) coloured trunks. They found more light coloured moths in woods with light trees and more dark coloured moths in woods with polluted trees. This data provides support for
    A. Artificial selection.
    B. Extinction.
    C. Inheritance of acquired characteristics.
    D. Natural selection.

25. Natural selection is
    A. The same thing as evolution.
    B. A possible but unproven mechanism of evolution.
    C. Only one of many possible mechanisms of evolution.
    D. The inheritance of acquired characteristics.

26. Scientists have found evidence to suggest that the first life appeared on earth approximately:
    A. 10 thousand years ago.
    B. 270 million years ago.
    C. 3 billion years ago.
    D. 6 billion years ago.
